# Smooth muscle 22 alpha protein inhibits VSMC foam cell formation by supporting normal LXRα signaling, ameliorating atherosclerosis

**DOI:** 10.1038/s41419-021-04239-w

**Published:** 2021-10-22

**Authors:** Dan-Dan Zhang, Yu Song, Peng Kong, Xin Xu, Ya-Kun Gao, Yong-Qing Dou, Lin Weng, Xiao-Wei Wang, Yan-Ling Lin, Fan Zhang, Hailin Zhang, Mei Han

**Affiliations:** 1 Department of Biochemistry and Molecular Biology, College of Basic Medicine, Key Laboratory of Medical Biotechnology of Hebei Province, Cardiovascular Medical Science Center, Hebei Medical University, Shijiazhuang, Hebei P.R. China; 2 College of Integrative Medicine, Hebei University of Chinese Medicine, Shijiazhuang, Hebei P.R. China; 3 Department of Laboratory of Lipid Metabolism, Institute of Basic Medicine, Hebei Medical University, Shijiazhuang, Hebei P.R. China; 4 Department of Pharmacology, College of Basic Medicine, Hebei Medical University, Shijiazhuang, Hebei P.R. China

**Keywords:** Lipoproteins, Actin, Proteomics, Atherosclerosis, Experimental models of disease

## Abstract

Vascular smooth muscle cells (VSMCs) are indispensable components in foam cell formation in atherosclerosis. However, the mechanism behind foam cell formation of VSMCs has not been addressed. We found a potential association between deletion of smooth muscle (SM) 22α and deregulated nuclear receptors liver X receptors (LXRs)/retinoid X receptor (RXR) signaling in mice. Here, we investigated the roles of SM22α in LXRα-modulated cholesterol homeostasis, and explore possible mechanisms underlying this process. We identified that the depletion of SM22α was a primary event driving VSMC cholesterol accumulation and the development of atherosclerosis in mice. Proteomic and lipidomic analysis validated that downregulation of SM22α was correlated with reduced expression of LXRα and ATP-binding cassette transporter (ABCA) 1 and increased cholesteryl ester in phenotypically modulated VSMCs induced by platelets-derived growth factor (PDGF)-BB. Notably, LXRα was mainly distributed in the cytoplasm rather than the nucleus in the neointimal and *Sm22α*^−/−^ VSMCs. Loss of SM22α inhibited the nuclear import of LXRα and reduced ABCA1-mediated cholesterol efflux via promoting depolymerization of actin stress fibers. Affinity purification and mass spectrometry (AP-MS) analysis, co-immunoprecipitation and GST pull-down assays, confocal microscopy, and stochastic optical reconstruction microscopy (STORM) revealed that globular-actin (G-actin), monomeric actin, interacted with and retained LXRα in the cytoplasm in PDGF-BB-treated and *Sm22α*^−/−^ VSMCs. This interaction blocked LXRα binding to Importin α, a karyopherin that mediates the trafficking of macromolecules across the nuclear envelope, and the resulting reduction of LXRα transcriptional activity. Increasing SM22α expression restored nuclear localization of LXRα and removed cholesterol accumulation via inducing actin polymerization, ameliorating atherosclerosis. Our findings highlight that LXRα is a mechanosensitive nuclear receptor and that the nuclear import of LXRα maintained by the SM22α-actin axis is a potential target for blockade of VSMC foam cell formation and development of anti-atherosclerosis.

## Introduction

Vascular smooth muscle cells (VSMCs) are a major cell type present at intimal thickenings and all stages of an atherosclerotic plaque but with altered phenotypes [1]. The phenotypic switching of VSMCs, which is characterized by reduced myofilament density and contractile protein expression, is a key event during the development of atherosclerosis [2, 3]. Recently, the relative contribution of VSMCs to total foam cell formation has been determined in human atherosclerosis and in the model of mice [4, 5]. However, the mechanism underlying phenotypically switched VSMCs transforming into foam cells remains unclear.

Liver X receptors (LXRs) are members of the nuclear receptor superfamily of ligand-activated transcription factors and are primarily located in the nuclei with or without bound ligand and regulate the expression of genes involved in cholesterol metabolism and inflammation in a tissue-specific manner [6, 7]. The intranuclear concentration of LXRs is maintained by a balance between nuclear import, nuclear export, and nuclear retention. The regulation of this balance provides important regulatory mechanisms for transcription [8]. LXRα-driven expression of the cholesterol efflux transporter ATP-binding cassette transporter (ABCA) 1 is essential for optimal reverse cholesterol transport in peripheral cells, which are associated with the pathogenesis of atherosclerosis [9]. VSMCs switched from contractile to synthetic phenotype metabolize lipids differently to contractile VSMCs, in part through decreased expression of ABCA1, resulting in an increased tendency to transform into foam cells [1, 6], but coordinated regulation of these processes has not been documented.

Smooth muscle (SM) 22α (also known as Transgelin), a differentiated VSMC marker [10], is involved in actin filament assembly and cytoskeletal rearrangements [11], which is required for maintaining the differentiated phenotype of VSMCs [12, 13]. The expression of SM22α has been demonstrated to be downregulated in atherosclerosis and neointima, and further resulted in the depolymerization of the F-actin cytoskeleton, which represents phenotypic changes of VSMCs from the contractile to the synthetic [14, 15]. Loss of SM22α in hypercholesterolemic *ApoE*-deficient mice results in increased atherosclerotic lesion area and a higher proportion of proliferating SMC-derived plaque cells [16], implying that there is a potential causal relationship between SM22α depletion and atherosclerotic lesion. We and others have demonstrated that disruption of SM22α enhances the inflammatory response in VSMCs [17–19]. Conversely, overexpression of SM22α inhibits proliferation, inflammation, and oxidative stress of VSMC via blockade of different upstream pathways, and prevents neointima hyperplasia and aortic aneurysm formation [20–22]. Furthermore, these physiological and pathological effects of SM22α are mediated by the regulation of actin dynamics to some extent [16, 17]. Recently, the aortic transcriptome profiling suggests that SM22α knockout (*Sm22α*^−/−^) mice exhibited the characteristics of pro-atherosclerosis with deregulated nuclear receptors LXR/RXR (retinoid X receptor) and atherosclerosis signaling pathways [20]. Thus, we hypothesized that the protective effects of SM22α on vascular homeostasis may involve the regulation of LXR signaling.

In the current study, we first demonstrated that nuclear accumulation of LXRα is regulated by SM22α-modulated actin dynamics, and altered actin dynamics by SM22α depletion and the resulting inhibition of LXRα nuclear import accelerate the transformation of VSMCs into foam cells and development of atherosclerosis. Monomeric actin (G-actin) from F-actin depolymerization disturbs LXRα nuclear import and retains it in the cytoplasm. The normal LXRα signaling supported by SM22α is a potential target for blockade of VSMC foam cell formation and development of anti-atherosclerosis.

## Results

### Depletion of SM22α contributes to the development of atherosclerosis in mice

**Fig. 1:**
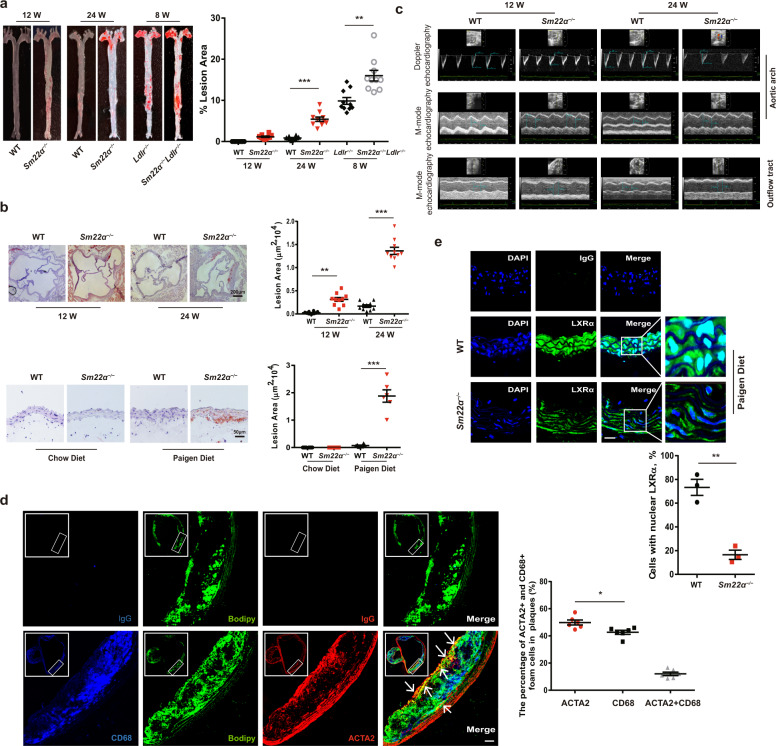
Impaired SM22α expression is associated with the development of atherosclerosis. **a**, **b** WT (*n* = 10) and *Sm22α*^−/−^ (*n* = 10) mice with or without *Ldlr*^−/−^ background (*n* = 10) fed Paigen diet for 8, 12 and 24 weeks respectively. Representative images of en face ORO-stained aortas (**a**), aortic sinus, aortic cross-sections (**b**) and quantification of lesion areas are shown. **c** M-mode and Doppler echocardiography images obtained from the aortic arch and outflow tract of WT (*n* = 15) and *Sm22α*^−/−^ (*n* = 15) mice fed Paigen diet for 12 and 24 weeks. A_s_: outflow tract and aortic diameter in systole; A_d_: outflow tract and aortic diameter in diastole. **d** Identification of SMC-derived foam cells within atherosclerotic lesion of *Sm22α*^−/−^ mice (*n* = 6) by CD68 (blue), ACTA2 (red) and Bodipy (green). Scale bar, 20 µm. Arrows indicated foam cells that were VSMCs-derived. **e** Representative immunofluorescence of LXRα (red) and quantification of cells with nuclear LXRα in the aortic sections from WT (*n* = 3) and *Sm22α*^−/−^ (*n* = 3) mice. Scale bar, 15 µm. Data and images are representative of at least three independent experiments. Data in (**a**) and (**b**) were analyzed by two-way and one-way ANOVA respectively. Data in (**d**) and (**e**) were analyzed by unpaired *t* test. *p < 0.05; **p < 0.01; ***p < 0.001.

We first took advantage of the published transcriptome analysis obtained on mouse aorta [23], to directly evaluate the sensitivity of *Sm22α*^−/−^ mice to atherosclerosis. The levels of serum total cholesterol (TC) and low-density lipoprotein cholesterol (LDL-C) gradually increased and were significantly higher in *Sm22α*^−/−^ mice than that in wild-type (WT) mice when fed the Paigen diet for 24 weeks with no change of serum triglyceride (TG) levels (Supplementary Fig. 1a–c). The diet significantly increased aortic cholesterol and cholesteryl ester (CE) content in *Sm22α*^−/−^ mice (Supplementary Fig. 1d). A positive oil red O staining was observed in aortic sinus sections of *Sm22α*^−/−^ but not of WT mice when fed the Paigen diet, accompanied with diffuse vascular wall thickening (Fig. 1a, b). Moreover, *Sm22α*^−/−^*Ldlr*^−/−^ mice on a Paigen diet displayed an aggravated atherosclerotic lesion compared to *Ldlr*^−/−^ mice (Fig. 1a and Supplementary Fig. 1e), consistent with the previous finding [16].

Aortic stiffness is believed to be the earliest detectable manifestation of adverse structural and functional changes within the aortic wall [24]. Compared with WT mice, the aortic stiffness parameters, including elastic modulus, stiffness index, and reverse/forward flow ratio, were obviously increased in the outflow tract and aortic arch of *Sm22α*^−/−^ mice fed Paigen diet, while aortic distensibility was decreased significantly compared with WT mice (Fig. 1c and Supplementary Fig. 1f). HE staining for the aortic sections also showed incomplete vascular structure and fragmentation of elastic fiber (Supplementary Fig. 1e) and a notable increase of fibrosis in the outflow tract and aortic arch of *Sm22α*^−/−^ mice fed Paigen diet for 12 weeks (Supplementary Fig. 1g, h), which was earlier than the atherosclerotic lesion. The expression of collagen I α (Col1α) increased and elastin (Eln) decreased in the aortic tissues of *Sm22α*^−/−^ mice (Supplementary Fig. 1i), suggesting that *Sm22α*^−/−^ mice develop an aortic stiffness phenotype.

Bodipy staining showed that 50 ± 2% of foam cells costained strongly with smooth muscle marker α-actin (ACTA2) (Fig. 1d). Cells expressing both ACTA2 and CD68 that is a marker for macrophages as a percentage of total CD68^+^ cells were 12 ± 2% (*n* = 6) in the atherosclerotic lesions of *Sm22α*^−/−^ mice (Fig. 1d), which was approximately one-fourth of the macrophages, consistent with that observed in the atherosclerotic lesions of *ApoE*^−/−^ mice and human coronary artery [5]. The expression of LXRα and ABCA1 at mRNA and protein levels decreased in the aortic tissues from *Sm22α*^−/−^ mice fed with Paigen diet or not, compared with WT mice (Supplementary Fig. 1j, k). Surprisingly, we observed numerous nuclear LXRα-staining negative VSMCs in the aortic sections from *Sm22α*^−/−^ mice (Fig. 1e), suggesting that LXRα nuclear localization is disturbed. Overall, these data demonstrated that SM22α depletion induces arteriosclerosis via a mechanism that involves the dysfunction of LXRα activity.

### Expression and activity of LXRα are abnormal in *Sm22α*^−/−^ VSMCs

**Fig. 2:**
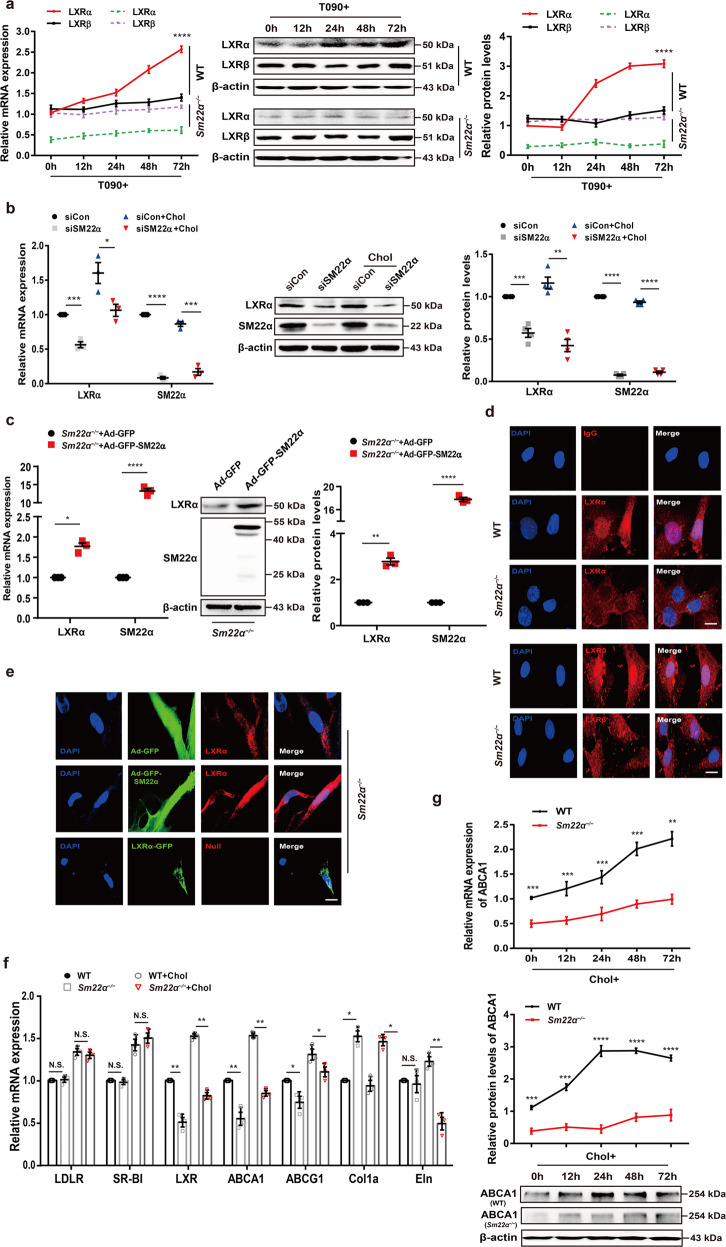

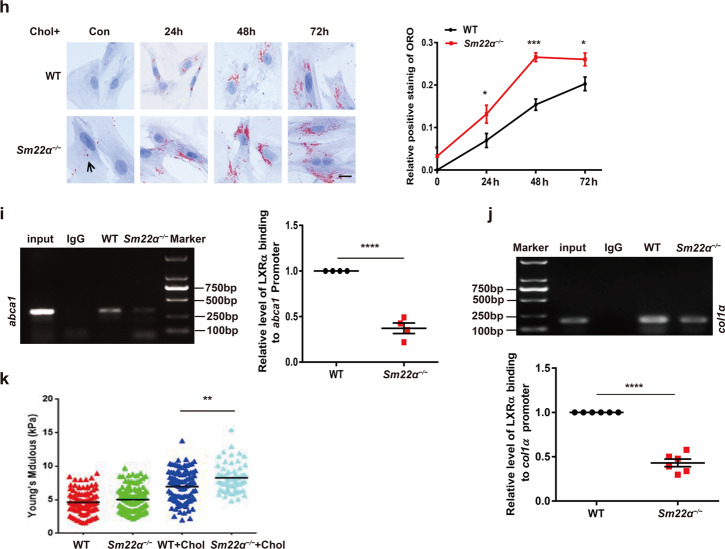
Expression and activity of LXRα is abnormal in *Sm22α*^−/−^ VSMCs. **a** qRT-PCR and Western blot analysis of LXRα and LXRβ in WT and *Sm22α*^−/−^ VSMCs treated with LXRs agonist T090 for 0, 12, 24, 48 and 72 h respectively (*n* = 3). **b** qRT-PCR and Western blot analysis of LXRα in WT VSMCs with or without cholesterol loading following knockdown of SM22α (*n* = 3). **c** qRT-PCR and Western blot analysis of LXRα and SM22α expression in *Sm22α*^−/−^ VSMCs transducted with Ad-GFP and Ad-GFP-SM22α for 24 h (*n* = 3). **d** Confocal microscopy images of LXRα and LXRβ distribution in WT and *Sm22α*^−/−^ VSMCs. Scale bar, 10 µm. **e** Immunofluorescence staining for endogenous LXRα and LXRα-GFP in *Sm22α*^−/−^ VSMCs transducted with Ad-GFP and Ad-GFP-SM22α or not. Scale bar, 10 µm. **f** qRT-PCR analysis of cholesterol intake (LDLR, SR-BI), efflux genes (ABCA1, ABCG1), and sclerosis-related genes (Col1α, Eln) in WT and *Sm22α*^−/−^ VSMCs incubated with or without cholesterol (*n* = 3). **g** The mRNA and protein levels of ABCA1 in WT and *Sm22α*^−/−^ VSMCs treated with cholesterol for 0, 12, 24, 48, and 72 h respectively (*n* = 3). **h** ORO staining of WT and *Sm22α*^−/−^ VSMCs stimulated with cholesterol for 0, 24, 48, and 72 h respectively and quantification of positive ORO staining. Scale bar, 20 μm. **i** The binding activity of LXRα to the promoter of *abca1* gene was decreased in *Sm22α*^−/−^ VSMCs (*n* = 4). **j** ChIP and RT-PCR detected LXRα binding to *col1α* promoter in WT and *Sm22α*^−/−^ VSMCs (*n* = 6). **k** The Young’s modulus of WT and *Sm22α*^−/−^ VSMCs treated with or without cholesterol (*n* = 120). Data and images are representative of at least three independent experiments. Data in (**a**), (**g**), and (**h**) were analyzed by Kruskal−Wallis rank-sum test and two-way ANOVA. Data in (**b**), (**c**), (**f**), (**i**), and (**j**) were analyzed by unpaired *t* test. NS not significantly different; *p < 0.05; **p < 0.01; ***p < 0.001; ****p < 0.0001.

We next mainly examined the effect of SM22α loss on the expression and distribution of LXRs in VSMCs as macrophages did not express SM22α [25]. The expression of LXRα was lower in *Sm22α*^−/−^ VSMCs than that in WT cells under basic conditions, and no difference in LXRβ expression was observed. T090- and cholesterol-induced LXRα expression was repressed in *Sm22α*^−/−^ VSMCs compared to WT VSMCs (Fig. 2a and Supplementary Fig. 2a). To further establish the causal relevance of SM22α in the expression of LXRα, WT VSMCs were transfected with the specific siRNA of SM22α and *Sm22α*^−/−^ VSMCs with Ad-GFP-SM22α. We showed that knockdown of SM22α resulted in reduced expression of LXRα at mRNA and protein levels in WT VSMCs with or without cholesterol loading (Fig. 2b). Conversely, the downregulation of LXRα was significantly reversed by the rescue of SM22α expression in *Sm22α*^−/−^ VSMCs (Fig. 2c).

As VSMCs with negative nuclear LXRα-staining were found in the aortic sections of *Sm22α*^−/−^ mice, we next determined whether the subcellular distribution of LXRα is changed in *Sm22α*^−/−^ VSMCs. We showed that endogenous LXRα was mainly accumulated in the nucleus of WT VSMCs. However, nuclear LXRα was almost little in *Sm22α*^−/−^ VSMCs, which exhibited diffuse cytoplasmic staining (Fig. 2d), and the ratio of cytoplasmic-/nuclear-LXRα protein markedly increased compared to WT controls (Supplementary Fig. 2b). The rescue of SM22α expression restored endogenous LXRα nuclear localization in *Sm22α*^−/−^ VSMCs (Fig. 2e), whereas the overexpression of LXRα-GFP was unable to do this, suggesting that LXRα nuclear accumulation is SM22α-dependent and not influenced by LXRα level, and that defect of LXRα nuclear localization further reduces its expression, as LXRα is also a direct target gene of LXRα and enhances its own expression [26].

Activation of LXRα is also able to suppress collagen expression not only promotes transcription of lipid transport and, metabolism genes [27, 28], and VSMCs, as the main origin of extracellular matrix (ECM), are a significant regulator of ECM remodeling and arterial stiffness [29]. We showed that the mRNA level of ABCA1, ABCG1, and Eln was significantly downregulated (Fig. 2f), while the expression of Col1α was significantly elevated in *Sm22α*^−/−^ VSMCs under basic and cholesterol loading conditions. By comparison, SM22α loss had little effect on the expression of cholesterol intake genes LDL receptor (LDLR) and scavenger receptor class B type I (SR-BI). Cholesterol-induced dynamic upregulation of ABCA1 expression observed from WT cells was missed in *Sm22α*^−/−^ VSMCs (Fig. 2g), accompanied by increased cholesterol accumulation in a time-dependent manner (Fig. 2h). Reduced ABCA1 expression and increased cholesterol accumulation were verified in VSMCs with knockdown of SM22α (Supplementary Fig. 2c–e). Chromatin immunoprecipitation (ChIP) assay showed that the binding activity of LXRα to the promoter of *abca1* and *col1α* genes was decreased in *Sm22α*^−/−^ VSMCs compared to WT VSMCs (Fig. 2i, j), in accordance with decreased ABCA1 and increased Col1α expression. Rescue of SM22α expression in *Sm22α*^−/−^ VSMCs by transduction with Ad-GFP-SM22α increased in binding activity of LXRα to *col1α* promoter with a reduced Col1α expression (Supplementary Fig. 2f, g). Overall, these data indicate that LXRα transcriptional regulatory activity is repressed in *Sm22α*^−/−^ VSMCs.

Not only does cholesterol deposits in foam cells at the atherosclerotic plaque, it also regulates cellular mechanics in atherosclerosis progression [30]. Next, the force-curve and Young’s modulus of individual VSMCs were measured by atomic force microscopy (AFM). E-modulus of *Sm22α*^−/−^ VSMCs was higher and significantly increased in response to cholesterol loading compared with WT cells (Fig. 2k), suggesting that cholesterol accumulation and substrate stiffness induce alternation of the biomechanics of VSMCs. Taken together, these results suggest that SM22α loss contributes to stiffness and foam cell formation of VSMCs.

### Nuclear localization and signaling of LXRα are impaired in phenotypically switched VSMCs

**Fig. 3:**
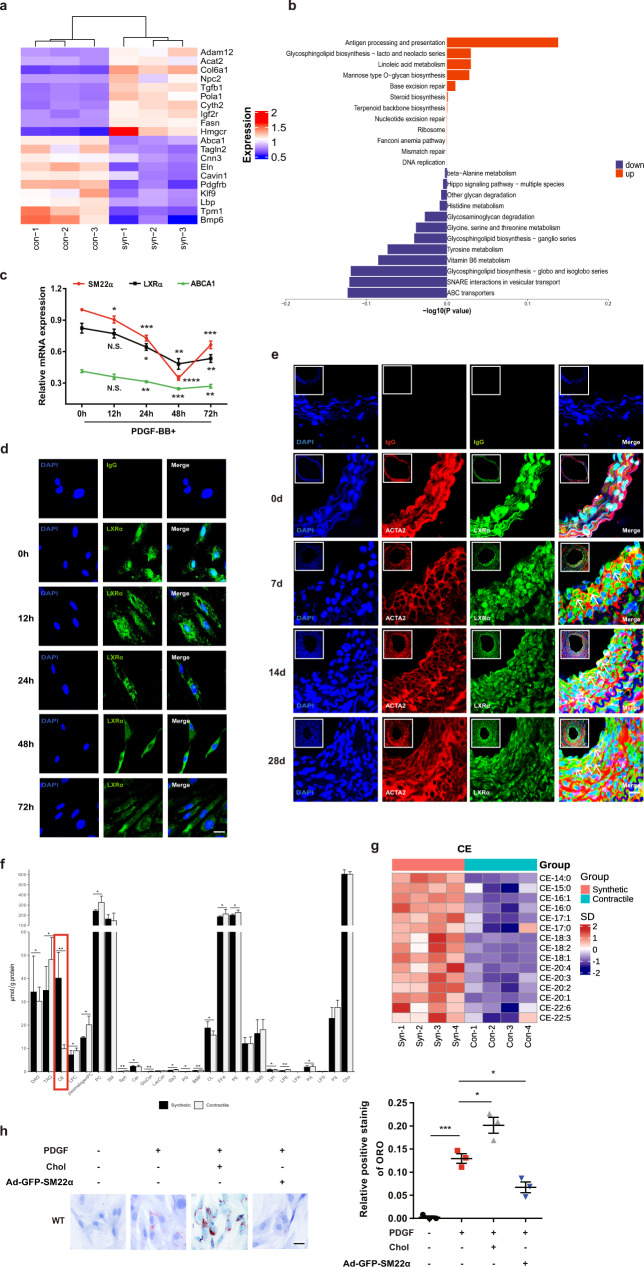

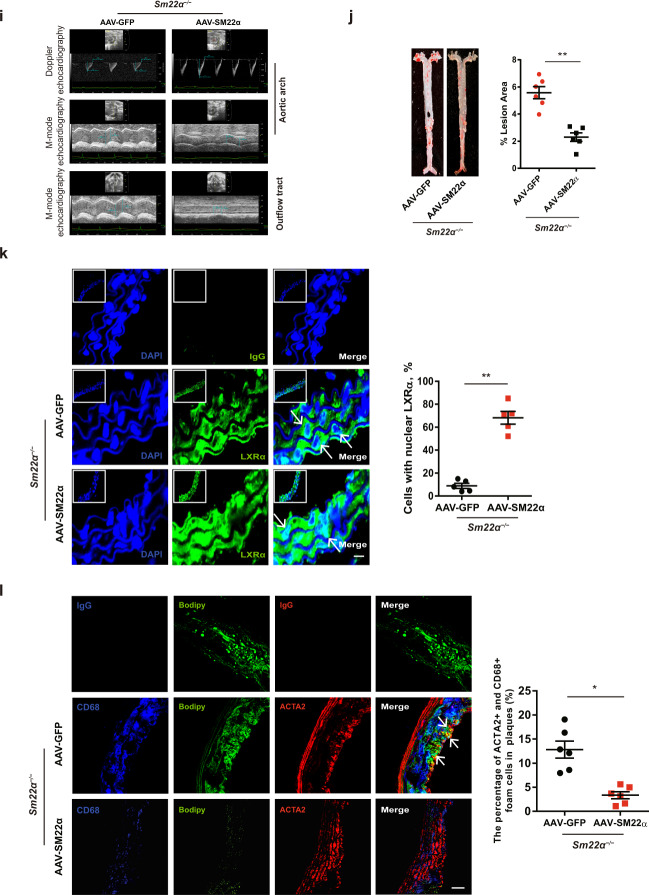
Function of the LXR.α-ABCA1 axis is impaired in phenotypically switched VSMCs. **a** Heatmap of proteomic analysis between synthetic and contractile VSMCs. **b** Analysis of KEGG pathway enriched by differentially expressed genes of proteomic analysis between synthetic and contractile VSMCs. **c** The mRNA expression of SM22α, LXRα, and ABCA1 in WT VSMCs treated with PDGF-BB for 0, 12, 24, and 48 h respectively (*n* = 3). **d** Confocal microscopy images of LXRα distribution in WT VSMCs incubated with PDGF-BB for 0, 12, 24, and 48 h respectively. Scale bar, 10 µm. **e** Confocal microscopy images of LXRα and ACTA2 in arterial walls of WT mice after ligation for 0, 7, 14, and 28 days. Scale bar, 20 µm. **f** Quantification of each lipid class in synthetic and contractile VSMCs. Lipid classes were expressed as μmol per g protein. **g** Heatmap of CEs between synthetic and contractile VSMCs. **h** ORO staining of WT VSMCs transducted with or without Ad-GFP-SM22α following with PDGF-BB and/or cholesterol treatment and quantification of positive ORO staining. Scale bar, 20 μm (*n* = 3) **i** M-mode and Doppler echocardiography images obtained from aortic arch and outflow tract of *Sm22α*^−/−^ mice transducted with AAV-GFP (*n* = 10) and AAV-SM22α (*n* = 10) fed Paigen diet for 12 weeks. A_s_: outflow tract and aortic diameter in systole; A_d_: outflow tract and aortic diameter in diastole. **j** Representative images of en face ORO-stained aortas and quantification of lesion areas (*n* = 6). **k** Representative immunofluorescence of LXRα (green) and quantification of cells with nuclear LXRα in the aortic sections from *Sm22α*^−/−^ mice transducted with AAV-GFP (*n* = 4) and AAV-SM22α (*n* = 4) fed Paigen diet for 24 weeks. Scale bar, 10 µm. Arrows indicated the distribution of LXRα. **l** Identification of SMC-derived foam cells within atherosclerotic lesion of *Sm22α*^−/−^ mice infected with AAV-GFP (*n* = 4) and AAV-SM22α (*n* = 4) fed Paigen diet for 24 weeks by CD68 (blue), ACTA2 (red) and Bodipy (green). Scale bar, 25 µm. Arrows indicated foam cells that were VSMCs-derived. Data and images are representative of at least three independent experiments. Data in (**c**) were analyzed by two-way ANOVA. Data in (**f**), (**h**), (**j**), (**k**) and (**l**) were analyzed by unpaired *t* test. *p < 0.05; **p < 0.01; ***p < 0.001; ****p < 0.0001.

SM22α loss is a prominent marker of phenotypic switching of VSMCs [10], and *Sm22α*^−/−^ VSMCs have the characteristic of synthetic and pro-inflammatory phenotypes [17–19]. To determine whether the dysfunction of the LXRα-ABCA1 axis is common in phenotypically modulated VSMCs, we performed the proteomic analysis of the contractile and synthetic VSMCs and showed that the expression of ABCA1 was markedly reduced in the synthetic VSMCs (Fig. 3a, b and Supplementary Fig. 3a). We validated that the expression of SM22α, ABCA1 and LXRα mRNAs decreased in a time-dependent manner in PDGF-BB-induced VSMCs (Fig. 3c). Notably, LXRα was gradually shifted from the nuclear to the cytoplasm in some VSMCs with extended PDGF-BB stimulation time (Fig. 3d). To determine whether nuclear localization of LXRα is impaired in modulated VSMCs in vivo, we examined VSMCs in the media and the neointima of mice. As expected, LXRα was mainly distributed in the nucleus of the medial VSMCs in the normal artery, whereas LXRα localization was shifted from the nucleus to the cytoplasm and merged with ACTA2 in the neointima VSMCs undergoing phenotypic modulation (Fig. 3e), indicating that LXRα nuclear localization was disturbed in these modulated VSMCs. To further confirm the effect of reduced LXRα-ABCA1 activity on lipid metabolism, the lipid profiles of VSMCs treated with PDGF-BB were assessed by a lipidomic analysis. Comparison of the lipidome between the contractile and synthetic phenotypes showed that CE was significantly increased compared with other lipids (Fig. 3f and Supplementary Fig. 3b), which was a prominent feature of the lipid profiles of synthetic VSMCs (Fig. 3g). Moreover, increased cholesterol deposition was also validated in PDGF-BB-induced VSMCs by ORO staining (Fig. 3h). Overall, these data further suggest that nuclear localization and signaling of LXRα are impaired during phenotypic switching of VSMCs, associated with the depletion of SM22α.

### Targeting SM22α supports normal LXRα signaling and ameliorates atherosclerosis

To further identify a potential causative link between SM22α expression and LXRα signaling, we selected an AAV carrying SM22α to perform a gain-of-function study in *Sm22α*^−/−^ mice fed Paigen diet. As expected, the administration of AAV-SM22α restored SM22α expression in the aortic wall of *Sm22α*^−/−^ mice accompanied with enhanced aortic GFP signal, indicating a high efficiency of viral transfection (Supplementary Fig. 3c, e). Compared with AAV-GFP mice, the administration of AAV-SM22α significantly reduced Paigen diet-induced aortic stiffness and atherosclerotic lesion (Fig. 3i, j and Supplementary Fig. 3d, e), indicating that AAV-SM22α intervention is effective. qRT-PCR showed that Col1α expression was decreased and the expression of Eln, LXRα and ABCA1 was elevated in the aortic wall of *Sm22α*^−/−^ mice with AAV-SM22α (Supplementary Fig. 3f), accompanied with reduced expression of MCP-1, MMP2, MMP9, VCAM-1 and ICAM-1 that are downstream of NF-κB, as LXRα has been reported to inhibit NF-κB activity [31]. Importantly, LXRα was mainly accumulated in the nucleus of VSMCs (Fig. 3k), and VSMC marker positive foam cells were reduced in the aortic arch of *Sm22α*^−/−^ mice with AAV-SM22α (Fig. 3l). Collectively, these findings suggest that SM22α ameliorates atherosclerosis via supporting nuclear localization of LXRα.

### Nuclear import of LXRα is regulated by actin dynamics

**Fig. 4:**
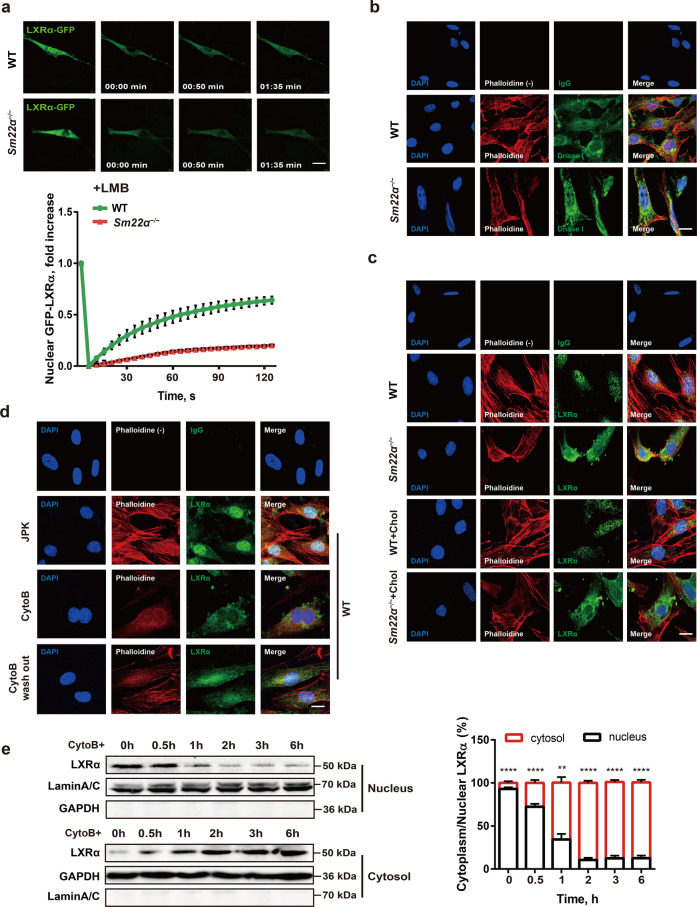
Nuclear import of LXRα is regulated by actin dynamics. **a** Fluorescence recovery after photobleaching (FRAP) studies with LXRα-GFP to measure nuclear import. Cells were pretreated with LMB. Decreased accumulation of nuclear fluorescence indicates a lower rate of nuclear import of LXRα-GFP in *Sm22α*^−/−^ VSMCs relative to WT controls (*n* = 25). **b** Representative images of F-actin (phalloidin, red) and G-actin (DnaseI, green) in WT and *Sm22α*^−/−^ VSMCs. Scale bar, 10 μm. **c**, **d** Representative images for F-actin (phalloidin, red) and LXRα (green) in WT and *Sm22α*^−/−^ VSMCs with cholesterol loading or not (**c**) and in WT VSMCs treated with JPK, CytoB and after CytoB washout (**d**). Scale bars, 10 µm. **e** Western blot analysis of cytoplasmic and nuclear LXRα in WT VSMCs treated with CytoB at different time points (*n* = 6). Data and images represent at least three independent experiments. Statistical analyses, unpaired *t* test and Kruskal−Wallis rank-sum test. **p < 0.01; ****p < 0.0001.

To elucidate the mechanism underlying cytoplasmic retention of LXRα, nuclear import of LXRα-GFP was measured by fluorescence loss in photobleaching (FLIP) and fluorescence recovery after photobleaching (FRAP) experiments. In WT cells, nuclear import of LXRα-GFP was extremely rapid, being effectively complete within 2 min (Fig. 4a and Supplementary Movie 1) and dramatically delayed and reduced in *Sm22α*^−/−^ cells (Supplementary Movie 2). LXRα phosphorylation and the formation of a heterodimer with retinoid X receptor (RXR) have been demonstrated to be required for LXRα nuclear accumulation [32, 33]. We showed that SM22α loss did not affect the phosphorylation of LXRα (Supplementary Fig. 4a), and also had no effect on the interaction between LXRα and RXRα (Supplementary Fig. 4b).

To investigate the possible sequestering proteins that retard LXRα nuclear import, we performed affinity purification using an anti-LXRα antibody, and the precipitates were subjected to mass spectrometry analysis. A total of 48 proteins potentially interacting with LXRα was identified (Supplementary Table). Next, we used GO enrichment analysis to cluster and characterize these proteins according to their biological processes (Supplementary Fig. 4c). Ultimately, the actin associated with muscle cell differentiation was selected as a candidate for its reported function in guiding transcriptional factors nuclear transport [34, 35]. As disruption of SM22α promotes actin cytoskeleton remodeling in VSMCs [17, 22], we subsequently compared actin organization between *Sm22α*^−/−^ and WT cells. *Sm22α*^−/−^ VSMCs exhibited less fraction of bundled stress fibers (Fig. 4b), and a higher total G-actin and parallel reduced ratio of F-actin to G-actin compared to WT cells (Supplementary Fig. 4d). Moreover, LXRα was mainly distributed in the perinuclear area of the cytoplasm with less F-actin in *Sm22α*^−/−^ VSMCs (Fig. 4c). To validate whether altered actin organization is associated with impaired nuclear translocation of LXRα, VSMCs were treated with jasplakinolide (JPK) which stabilizes F-actin and cytochalasin-B (CytoB) that inhibits actin polymerization. We showed that LXRα almost entirely remained in the cytoplasm of VSMCs treated by CytoB (Fig. 4d), with a decreased ratio of F-actin to G-actin (Supplementary Fig. 4d), and JPK did not affect the nuclear accumulation of LXRα. CytoB washout restored actin dynamics and distribution of LXRα in the nucleus (Fig. 4d and Supplementary Fig. 4d). Western blot for LXRα expression in the nuclear and cytoplasm fractions showed that LXRα protein was gradually shifted from the nuclear to the cytoplasm during CytoB treatment for different times, with increased cytoplasm/nucleus ratio of LXRα (Fig. 4e). Together, these data for the first time demonstrated that LXRα is a mechanosensitive nuclear receptor and that nuclear translocation of LXRα is regulated by actin dynamics.

### G-actin directly interacts with and retains LXRα in the cytoplasm

**Fig. 5:**
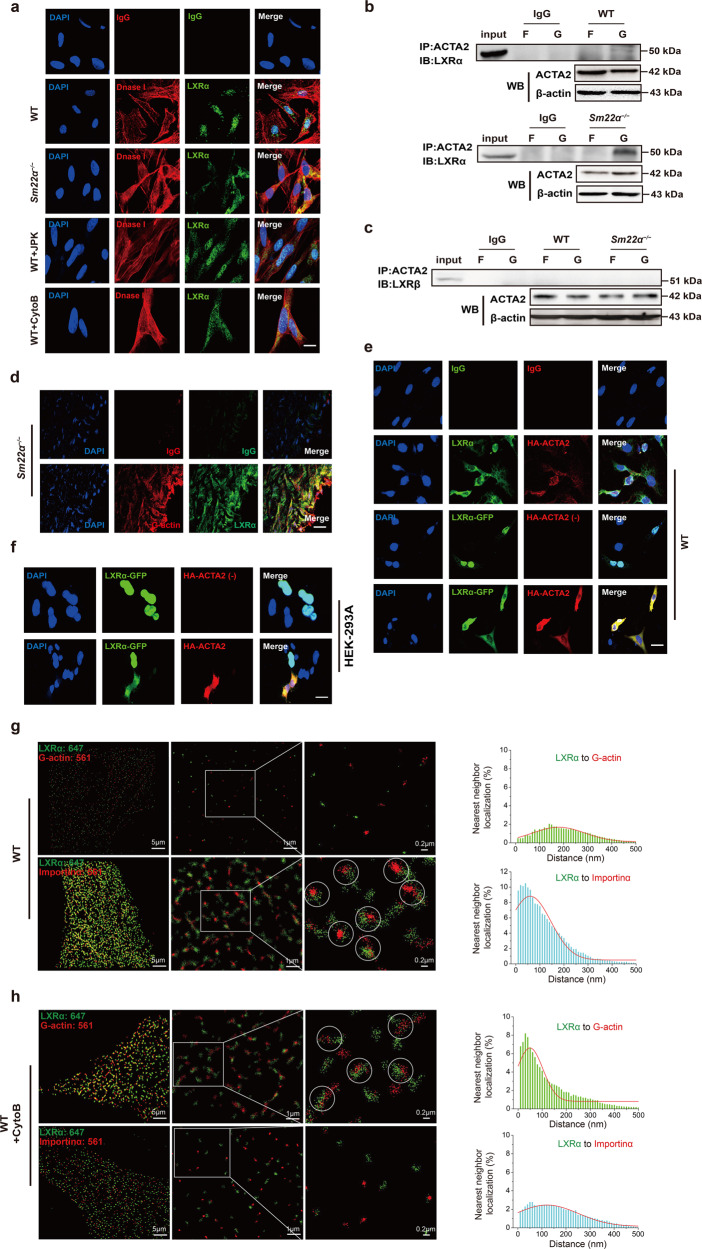

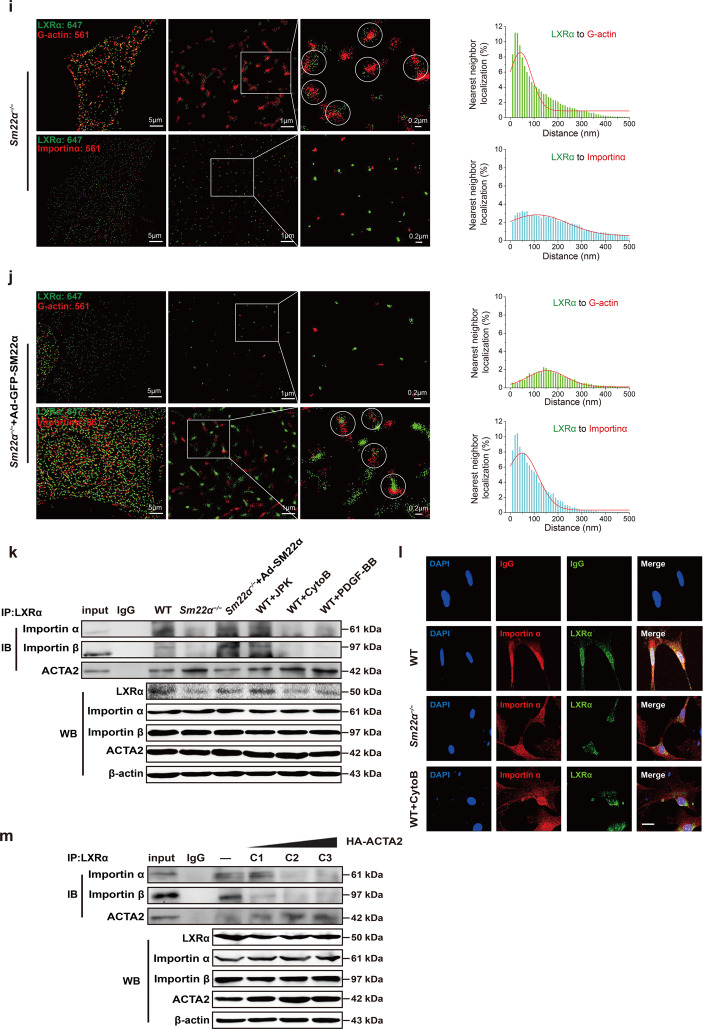
G-actin interacts with and retains LXRα in the cytoplasm, blocking LXRα binding to Importin α. **a** Double immunofluorescence staining for G-actin (DnaseI, red) and LXRα (green) in WT VSMCs accompanied with the treatment of JPK or CytoB and also in *Sm22α*^−/−^ VSMCs. Scale bar, 10 μm. **b**, **c** Co-immunoprecipitation of ACTA2 and LXRα (**b**) and LXRβ (**c**) respectively in F- and G-actin fractions of WT and *Sm22α*^−/−^ VSMCs (*n* = 3). **d** Double immunofluorescence staining of G-actin (Dnase1, red) and LXRα (green) or IgG in the atherosclerotic lesion in the aortic wall of *Sm22α*^−/−^ mice. Scale bar, 20 μm. **e** Representative immunofluorescence staining for endogenous LXRα (green) and LXRα-GFP (green) in WT VSMCs transfected with HA-ACTA2 (red, stained by anti-HA antibody) or not. Scale bar, 15 μm. **f** Representative immunofluorescence staining for LXRα-GFP (green) and HA-ACTA2 (red, stained by anti-HA antibody) in HEK-293A cells. Scale bar, 10 μm. **g**−**j** Two-color STORM images and quantification of the colocalization degree between LXRα and G-actin as well as Importin α in WT VSMCs with (**h**) or without (**g**) CytoB treatment and *Sm22α*^−/−^ VSMCs with (**j**) or without (**i**) Ad-GFP-SM22α infection (*n* > 10). **k** Co-immunoprecipitation of LXRα and Improtin α, Improtin β or ACTA2 in WT and *Sm22α*^−/−^ V*S*MCs with or without JPK, CytoB, PDGF-BB and Ad-GFP-SM22α treatment (*n* = 3). **l** Double immunofluorescence staining for Importin α (red) and LXRα (green) in WT and *Sm22α*^−/−^ V*S*MCs as well as CytoB-treated WT VSMCs. Scale bar, 15 μm. **m** Co-immunoprecipitation of LXRα and Importin α, Improtin β or ACTA2 in WT VSMCs transfected with HA-ACTA2 of different concentrations (*n* = 3). Data and images represent at least three independent experiments.

To test whether LXRα directly interacts with actin in vivo, we performed computational docking analysis for G-actin and LXRα via the ZDOCK server and discovered the highest-scored predicted model of interaction between them (Supplementary Fig. 4e). As LXRα did not colocalize with the F-actin cytoskeleton (Fig. 4c), we used DnaseI to label G-actin and observed that G-actin colocalized with LXRα in the cytoplasm of *Sm22α*^−/−^ and CytoB-treated VSMCs (Fig. 5a). Next, F-actin and G-actin fractions isolated from WT and *Sm22α*^−/−^ VSMCs were subjected to co-immunoprecipitation (co-IP) with specific anti-ACTA2 and anti-LXRα antibodies. A specific LXRα band was present in the complex immunoprecipitated by the anti-ACTA2 antibody in the G-actin fraction but not in the F-actin fraction of *Sm22α*^−/−^ but not WT VSMCs (Fig. 5b). Meanwhile, ACTA2 did not co-immunoprecipitate with LXRβ in both F-actin and G-actin fractions of *Sm22α*^−/−^ VSMCs (Fig. 5c). Importantly, an atherosclerotic lesion assay using DNaseI and anti-LXRα antibodies revealed a colocalization between endogenous LXRα and G-actin in the aortic wall of *Sm22α*^−/−^ mice (Fig. 5d). To further validate that increased G-actin level contributes to the retention of LXRα in the cytoplasm, we overexpressed HA-ACTA2 in WT VSMCs and showed that DNaseI-stained G-actin markedly increased (Supplementary Fig. 5a). Importantly, endogenous LXRα redistributed from the nucleus to the cytoplasm and colocalized with ACTA2 in the VSMCs (Fig. 5e), and exogenous LXRα-GFP was so in co-expressed cells. Similar results were observed in HEK-293A cells co-expressing LXRα-GFP and HA-ACTA2, different from the cells expressing LXRα-GFP alone that had nuclear LXRα-GFP fluorescence (Fig. 5f). Thus, G-actin is a novel inhibitor of LXRα nuclear accumulation.

To gain direct insight into G-actin-LXRα interaction in cells, we exploited stochastic optical reconstruction microscopy (STORM) [36], a super-resolution imaging method, to examine the spatial distributions of the two proteins. In WT cells, an obvious much broader peak was observed for the nearest-neighbor distribution of LXRα and G-actin (Fig. 5g). Upon treatment with CytoB, STORM displayed a significant reduction in the nearest-neighbor distance between LXRα and G-actin (Fig. 5h). A quantitatively similar increase in colocalization between LXRα and G-actin was observed using *Sm22α*^−/−^ VSMCs (Fig. 5i). Such colocalization was abolished by the rescue of SM22α expression (Fig. 5j). Together, these findings suggest that LXRα is recruited by G-actin.

### G-actin binding disturbs interaction between LXRα and Importin α

Binding to Importin α that is to serve as an adaptor is the first step in the nuclear transport of nuclear receptors by that link them to Importin β to form a ternary complex in the cytoplasm [37]. We observed that neither CytoB treatment nor loss of SM22α changed Importin α expression (Fig. 5k). Notably, the expression of Importin α in the LXRα-immunoprecipitated complex decreased evidently or even disappeared in *Sm22α*^−/−^ VSMCs compared to WT cells, which was returned the level to WT cells via the rescue of SM22α expression (Fig. 5k). Similarly, the LXRα-Importin α complex was decreased in CytoB and PDGF-BB-treated WT VSMCs, accompanied with reduced nuclear LXRα expression (Supplementary Fig. 5b), indicating that Importin α-mediated LXRα nuclear import was inhibited by altered actin dynamics, associated with VSMC phenotypes. These results were verified by immunofluorescence staining and confocal analysis (Fig. 5l). Next, we used STORM to examine the spatial relationship of LXRα to Importin α. In WT VSMCs, LXRα colocalized with Importin α, which was reflected by a nearest-neighbor distribution with a sharp peak near the 10−50 nm theoretical resolution limit of STORM (Fig. 5g), whereas disruption of F-actin by CytoB treatment or SM22α knockout completely eliminated this natural colocalizations (Fig. 5h, i), which displayed increased dramatically the nearest-neighbor distribution. The degree of colocalization between LXRα and Importin α was similar to WT cells upon the rescue of SM22α expression (Fig. 5j). This finding was supported by co-immunoprecipitation experiments (Fig. 5m). Taken together, these results suggest that G-actin acts as a molecular shield against LXRα binding to Importin α.

### The C-terminal domain mediates the interaction between G-actin and LXRα

**Fig. 6:**
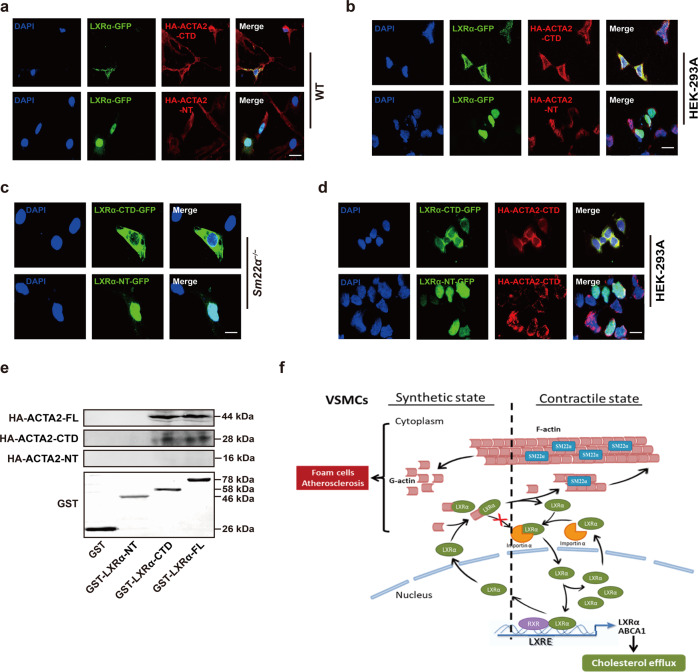
The C-terminal domain mediates the interaction between G-actin and LXRα. **a** Representative immunofluorescence staining for LXRα-GFP (green) in WT VSMCs transfected with HA-ACTA2-CTD (red) or HA-ACTA2-NT (red). Scale bar, 15 μm. **b** Representative immunofluorescence staining for LXRα-GFP (green) and HA-ACTA2-CTD (red) or HA-ACTA2-NT (red) in HEK-293A cells. Scale bar, 15 μm. **c** LXRα-CTD-GFP (green) or LXRα-NT-GFP (green) was transfected into *Sm22α*^−/−^ VSMCs. Scale bar, 10 μm. **d** LXRα (-CTD, -NT)-GFP (green) and HA-ACTA2-CTD (red) were co-expressed in HEK-293A cells. Scale bar, 10 μm. **e** Interaction of HA-ACTA2 (-FL, -CTD, -NT) and GST-LXRα (-FL, -CTD, -NT) proteins analyzed by in vitro pull-down assay (*n* = 3). **f** Schematic representation of a working model in which SM22α inhibits VSMC-derived foam cell formation by blocking actin-LXRα signaling ameliorating atherosclerosis. Data and images represent at least three independent experiments.

To characterize which part of ACTA2 is responsible for LXRα interaction, we then reconstructed the two structural domains of ACTA2 with HA-tagged truncated N-terminus (HA-ACTA2-NT, aa. 1−140) and C-terminal domains (HA-ACTA2-CTD, aa. 141−377) (Supplementary Fig. 6a), and used different truncation derivatives of ACTA2 to gain insight into the location of the interaction. The cells expressing HA-ACTA2-CTD displayed increased LXRα-GFP fluorescence intensity in the cytoplasm and reduced nuclear localization of LXRα-GFP (Fig. 6a) like HA-ACTA2-overexpressed cells (Fig. 5f). Furthermore, the ACTA2-CTD, but not ACTA2-NT, colocalized with LXRα-GFP in the cytoplasm. Similarly, in HEK293A cells co-expressing LXRα-GFP and ACTA2 truncation derivatives, the ACTA2-CTD colocalized with LXRα-GFP in the cytoplasm and abolished nuclear LXRα-GFP, whereas the ACTA2-NT did not influence the nuclear accumulation of LXRα-GFP (Fig. 6b), suggesting that ACTA2-CTD predominantly contributed to this interaction and retarded nuclear import of LXRα.

Because there were no RPEL motifs that bind to actin in LXRα sequence [38], in turn, we constructed the two truncated mutants of LXRα-N-terminus (NT, aa. 1−170) that contained the DNA binding domain and three nuclear localization sequences (NLSs) (NLS1, 2 and 4) and LXRα-C-terminal domains (CTD, aa. 171−445) that included the hinge region, one NLS (NLS3) and the putative ligand-binding domain (Supplementary Fig. 6b) [8, 39], and transfected them into *Sm22α*^−/−^ VSMCs. We showed that only LXRα-NT was accumulated in the nucleus, and LXRα-CTD, like endogenous LXRα, was also trapped in the cytoplasm of *Sm22α*^−/−^ cells (Fig. 6c). Similarly, nuclear localization of only LXRα-NT was observed in HEK293A cells co-expressing HA-ACTA2-CTD, whereas LXRα-CTD colocalized with ACTA2-CTD in the cytoplasm (Fig. 6d). Next, peptide pull-down experiments with recombinant purified GST-LXRα, GST-LXRα-NT, and GST-LXRα-CTD revealed that both GST-LXRα and GST-LXRα-CTD bound directly to ACTA2 and ACTA2-CTD rather than ACTA2-NT (Fig. 6e). Thus, LXRα-CTD is the region for ACTA2 recognizing and binding to LXRα. Together, these data suggest that the C-terminal domains mediate the interaction between G-actin and LXRα.

## Discussion

In the present study, we showed that the depletion of SM22α dysregulated LXRα signaling and promoted foam cell formation of VSMCs and the development of atherosclerosis. G-actin interacted with LXRα and inhibited its nuclear import, as the complex blocked LXRα binding to Imporin α. SM22α regulated the nuclear localization of LXRα through a mechanism in which F-actin polymerization by SM22α led to dissociation of this complex (Fig. 6f). Using *Sm22α*^−/−^ and *Sm22α*^−/−^*Ldlr*^−/−^mice, we provide evidence for a causative role of SM22α loss in LXRα signaling and VSMCs foam cell formation. G-actin was identified as a negative regulator of the LXRα nuclear import and activity.

The disruption of LXRα is believed to be an important factor in the pathological development of atherosclerosis via leading to foam cell formation in macrophages of the arterial wall [9, 40, 41]. Though the expression of LXRα is lower in human VSMCs, limited studies have demonstrated that LXRα can influence proliferation, contractility, apoptosis, and calcification in VSMCs [42]. Moreover, ABCA1 expression is reduced in neointimal VSMCs compared with those isolated from the medial layer [43], more so in advanced relative to early atherosclerosis [44]. In the current study, similar atherosclerotic phenotypes to those of LXRα-deficient mice were observed in *Sm22α*^−/−^ mice in the context of hypercholesterolemia. The diffuse thickenings of the vascular walls and aortic stiffness existed in *Sm22α*^−/−^ mice on a Paigen diet for 12 weeks, which are widely considered the most likely precursor of atherosclerotic plaques [1, 45]. Rescue of SM22α expression could alleviate cholesterol overload and displayed anti-atherogenic effects that presented as reduced aortic stiffness and lesion area. Interestingly, we demonstrated that one different aspect from the study on *Lxrα*^−/−^ mice is that the increased atherosclerosis in *Sm22α*^−/−^ mice is associated with an inability of VSMCs rather than macrophages to efficiently efflux cholesterol through the LXR pathway. More importantly, the observation that nuclear import of LXRα was impaired in VSMCs of *Sm22α*^−/−^ mice was removed by the rescue of SM22α expression in vitro and in vivo. Our findings suggest a particularly important role for SM22α in LXRα-mediated cholesterol homeostasis and contractile phenotype in VSMCs especially in the context of hypercholesterolemia and provide evidence that SM22α contributes to the anti-atherogenic effects of LXRα on VSMCs.

De-differentiation, modulation or phenotype switching of VSMCs is characterized by reduced myofilament density and lower expression of contractile proteins [1]. It is known that modulated VSMCs predominate in the thickened arterial intima at atherosclerosis-prone sites prior to the onset of plaque formation and VSMC foam cell formation is resulted from modulated VSMCs engulfing oxidized low-density lipoprotein [46]. In the present study, the proteomic and lipidomic analysis showed that SM22α loss correlated with reduced LXRα-ABCA1 expression and increased cholesteryl ester in phenotypically modulated VSMCs. We validated that LXRα was redistributed from the nuclear to the cytoplasm in VSMCs upon PDGF-BB stimulation and in the neointima VSMCs and that LXRα colocalized with G-actin in the cytoplasm, suggesting that LXRα nuclear localization is regulated by actin dynamics and is impaired as a result of VSMC phenotypic switching. Our findings indicated that SM22α loss-mediated aberrant actin-LXRα signaling pathway guides modulated VSMCs to ultimately transform into foam cells. Our results support the idea that lipid metabolism programming is a critical event in phenotypic switching of VSMCs and that SM22α activates the LXRα-ABCA1 axis to maintain lipid homeostasis through the modulation of cytoskeletal actin polymerization.

We acknowledge several limitations of this study. First, SM22α is not only expressed in SMCs but also expressed in other lineages, such as cardiomyocytes during development and myeloid cells [47]. Therefore, using inducible SMC-specific SM22α knockout mice are warranted to accurately define a more definitive causal relationship between SM22α expression in VSMCs and their contribution to atherosclerotic lesion formation. Second, polymerization and depolymerization in live cells are regulated by actin-binding proteins. It needs further to explore whether and how other actin-binding proteins are involved in the regulation of LXRα signaling and cholesterol metabolism. Finally, given the involvement of the actin cytoskeleton remodeling in phenotypic switching of VSMC, it will be important to determine how these and similar pathways of aberrant actin-to-LXRα crosstalking can be intervened by the development of therapeutic agents.

## Supplementary information

Supplementary Materials

Supplementary figure 1

Supplementary figure 2

Supplementary figure 3

Supplementary figure 4

Supplementary figure 5

Supplementary figure 6

Fluorescence recovery after photobleaching (FRAP) studies with LXRα-GFP to measure nuclear import in WT VSMCs.

FRAP studies with LXRα-GFP to measure nuclear import in SM22α KO VSMCs.

## Data Availability

The data that support the findings of this study are available from the corresponding author upon reasonable request.
