# Correction: Smooth muscle 22 alpha protein inhibits VSMC foam cell formation by supporting normal LXRα signaling, ameliorating atherosclerosis

**DOI:** 10.1038/s41419-021-04360-w

**Published:** 2021-11-10

**Authors:** Dan-Dan Zhang, Yu Song, Peng Kong, Xin Xu, Ya-Kun Gao, Yong-Qing Dou, Lin Weng, Xiao-Wei Wang, Yan-Ling Lin, Fan Zhang, Hailin Zhang, Mei Han

**Affiliations:** 1 Department of Biochemistry and Molecular Biology, College of Basic Medicine, Key Laboratory of Medical Biotechnology of Hebei Province, Cardiovascular Medical Science Center, Hebei Medical University, Shijiazhuang, Hebei People’s Republic of China; 2 College of Integrative Medicine, Hebei University of Chinese Medicine, Shijiazhuang, Hebei People’s Republic of China; 3 Department of Laboratory of Lipid Metabolism, Institute of Basic Medicine, Hebei Medical University, Shijiazhuang, Hebei People’s Republic of China; 4 Department of Pharmacology, College of Basic Medicine, Hebei Medical University, Shijiazhuang, Hebei People’s Republic of China

**Keywords:** Lipoproteins, Actin, Proteomics, Atherosclerosis, Experimental models of disease

Correction to: *Cell Death and Disease* 10.1038/s41419-021-04239-w, published online 22 October 2021

The original version of this article unfortunately contained a mistake. Due to a typesetting error some of the figures were omitted and figure legends were misplaced. We sincerely apologize for the errors. The correct figures and legends can be found below.

**Fig. 1:**
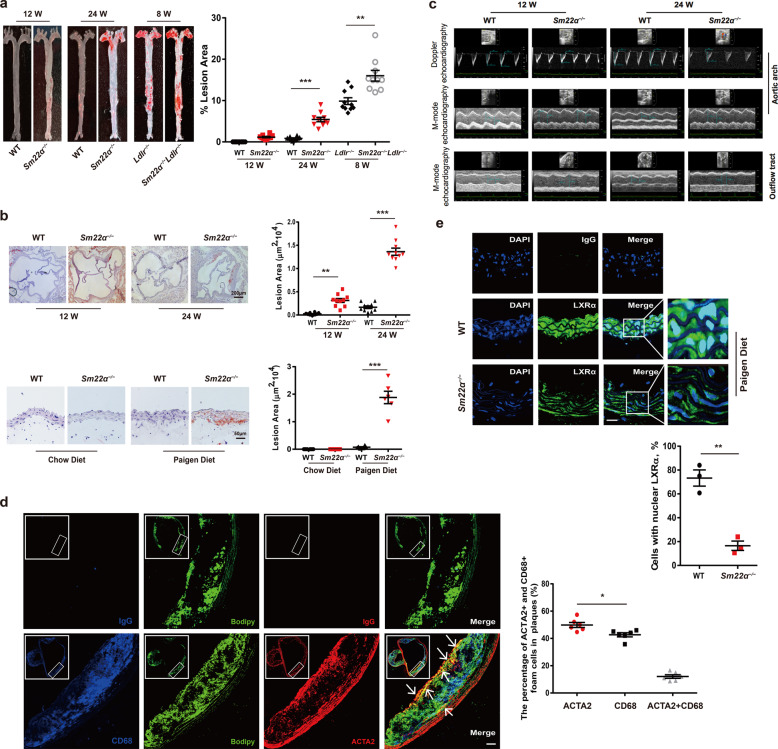
Impaired SM22α expression is associated with development of atherosclerosis. **a**, **b** WT (*n* = 10) and *Sm22α*^−/−^ (*n* = 10) mice with or without *Ldlr*^−/−^ background (*n* = 10) fed Paigen diet for 8, 12, and 24 weeks, respectively. Representative images of *en face* ORO-stained aortas (**a**), aortic sinus, aortic cross sections (**b**), and quantification of lesion areas are shown. **c** M-mode and Doppler echocardiography images obtained from aortic arch and outflow tract of WT (*n* = 15) and *Sm22α*^−/−^ (*n* = 15) mice fed Paigen diet for 12 and 24 weeks. *A*_s_: outflow tract and aortic diameter in systole; *A*_d_: outflow tract and aortic diameter in diastole. **d** Identification of SMC-derived foam cells within atherosclerotic lesion of *Sm22α*^−/−^ mice (*n* = 6) by CD68 (blue), ACTA2 (red), and Bodipy (green). Scale bar, 20 µm. Arrows indicated foam cells, which were VSMCs-derived. **e** Representative immunofluorescence of LXRα (red) and quantification of cells with nuclear LXRα in the aortic sections from WT (*n* = 3) and *Sm22α*^−/−^ (*n* = 3) mice. Scale bar, 15 µm. Data and images are representative of at least three independent experiments. Data in **a** and **b** were analyzed by two-way and one-way ANOVA, respectively. Data in **d** and **e** were analyzed by unpaired *t*-test. *p < 0.05; **p < 0.01; ***p < 0.001.

**Fig. 2:**
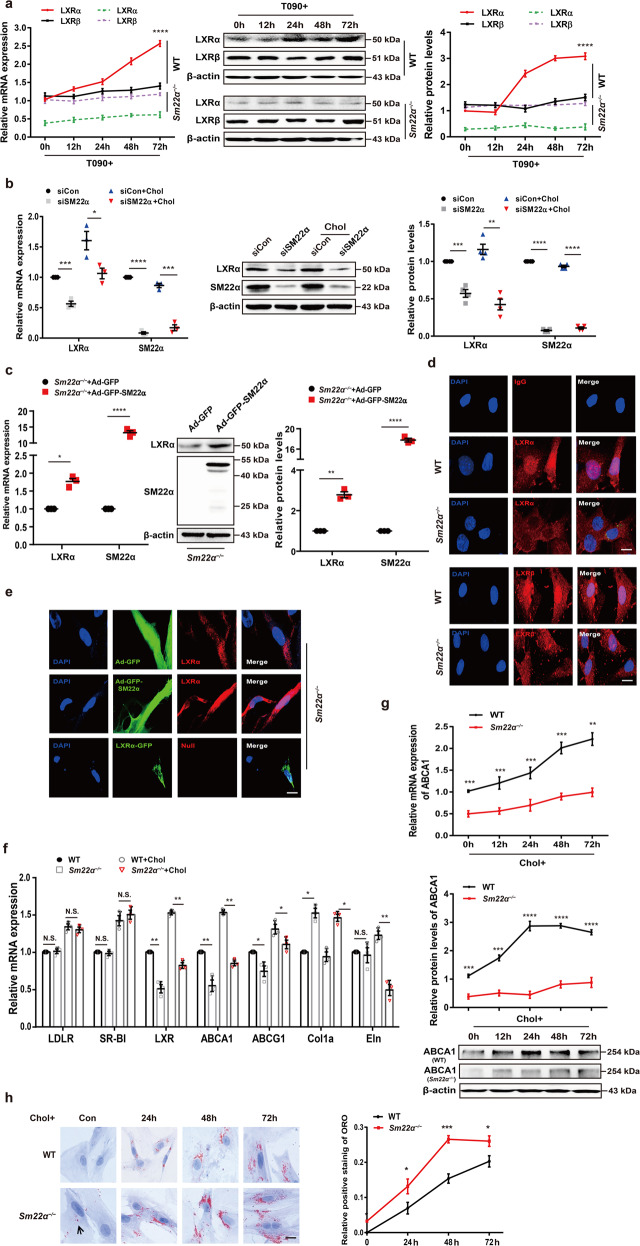

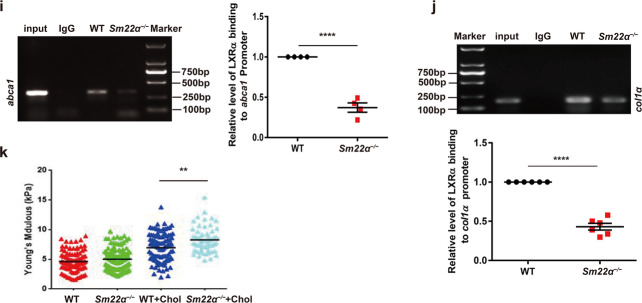
Expression and activity of LXRα is abnormal in *Sm22α*^−/−^ VSMCs. **a** qRT-PCR and Western blot analysis of LXRα and LXRβ in WT and *Sm22α*^−/−^ VSMCs treated with LXRs agonist T090 for 0, 12, 24, 48, and 72 h, respectively, (*n* = 3). **b** qRT-PCR and western blot analysis of LXRα in WT VSMCs with or without cholesterol loading following knockdown of SM22α (*n* = 3). **c** qRT-PCR and western blot analysis of LXRα and SM22α expression in *Sm22α*^−/−^ VSMCs transducted with Ad-GFP and Ad-GFP-SM22α for 24 h (*n* = 3). **d** Confocal microscopy images of LXRα and LXRβ distribution in WT and *Sm22α*^−/−^ VSMCs. Scale bar, 10 µm. **e** Immunofluorescence staining for endogenous LXRα and LXRα-GFP in *Sm22α*^−/−^ VSMCs transducted with Ad-GFP and Ad-GFP-SM22α or not. Scale bar, 10 µm. **f** qRT-PCR analysis of cholesterol intake (LDLR, SR-BI), efflux genes (ABCA1, ABCG1) and sclerosis related genes (Col1α, Eln) in WT and *Sm22α*^−/−^ VSMCs incubated with or without cholesterol (*n* = 3). **g** The mRNA and protein levels of ABCA1 in WT and *Sm22α*^−/−^ VSMCs treated with cholesterol for 0, 12, 24, 48, and 72 h, respectively (*n* = 3). **h** ORO staining of WT and *Sm22α*^−/−^ VSMCs stimulated with cholesterol for 0, 24, 48, and 72 h, respectively, and quantification of positive ORO staining. Scale bar, 20 μm. **i** The binding activity of LXRα to the promoter of *abca1* gene was decreased in *Sm22α*^−/−^ VSMCs (*n* = 4). **j** ChIP and RT-PCR detected LXRα binding to *col1α* promoter in WT and *Sm22α*^−/−^ VSMCs (*n* = 6). **k** The Young’s modulus of WT and *Sm22α*^−/−^ VSMCs treated with or without cholesterol (*n* = 120). Data and images are representative of at least three independent experiments. Data in **a**, **g**, and **h** were analyzed by Kruskal–Wallis rank sum test and two-way ANOVA. Data in **b**, **c**, **f**, **i**, and **j** were analyzed by unpaired *t*-test. N.S. not significantly different; *p < 0.05; **p < 0.01; ***p < 0.001; ****p < 0.0001.

**Fig. 3:**
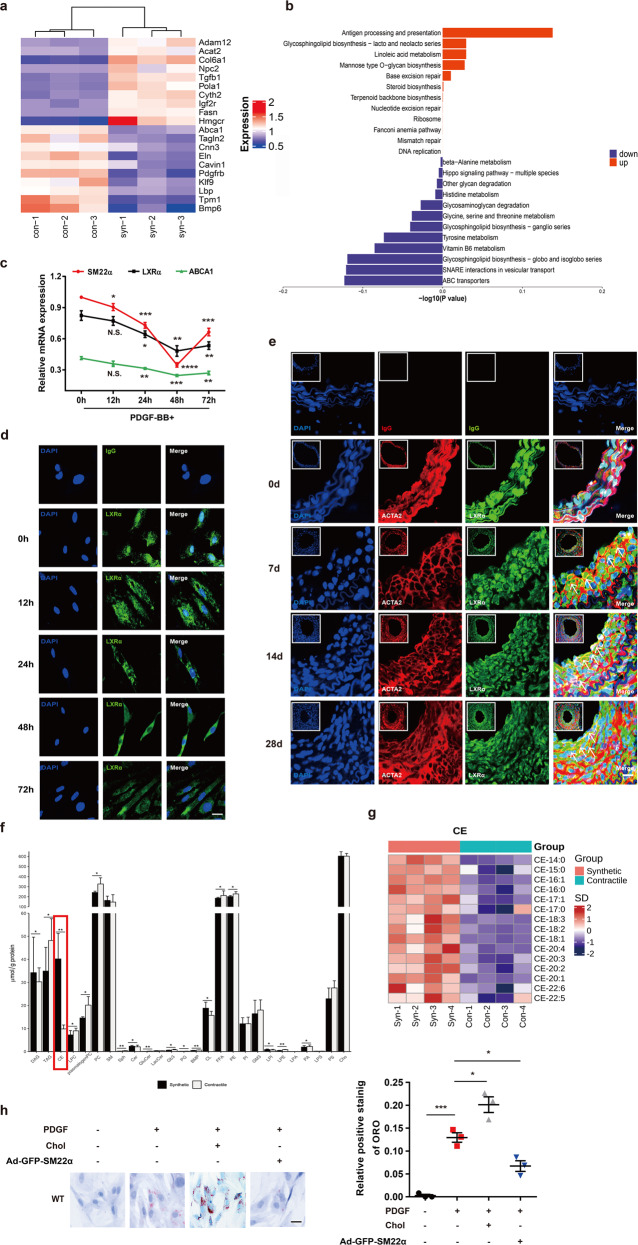

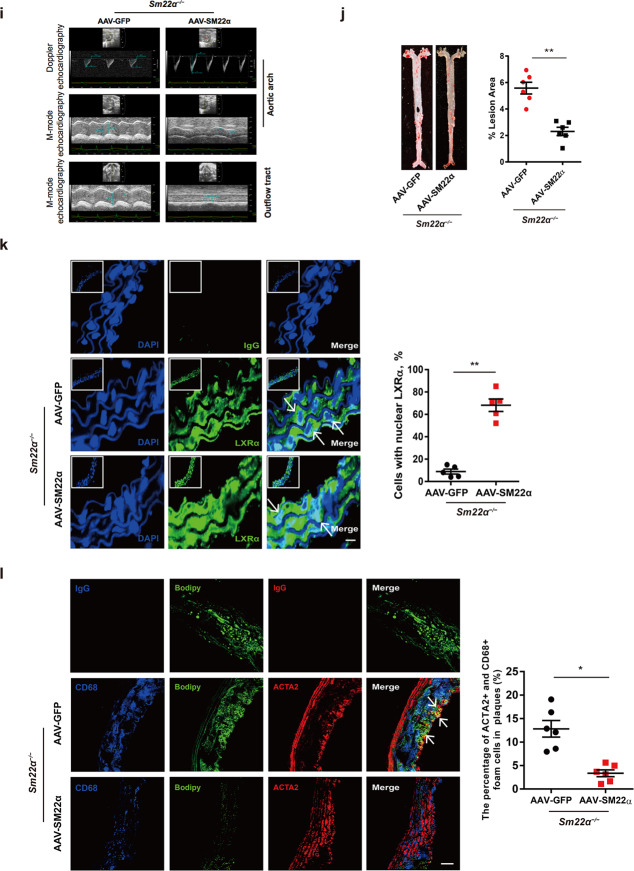
Function of LXRα-ABCA1 axis is impaired in phenotypically switched VSMCs. **a** Heatmap of proteomic analysis between synthetic and contractile VSMCs. **b** Analysis of KEGG pathway enriched by differentially expressed genes of proteomic analysis between synthetic and contractile VSMCs. **c** The mRNA expression of SM22α, LXRα, and ABCA1 in WT VSMCs treated with PDGF-BB for 0, 12, 24, and 48 h, respectively (*n* = 3). **d** Confocal microscopy images of LXRα distribution in WT VSMCs incubated with PDGF-BB for 0, 12, 24, and 48 h, respectively. Scale bar, 10 µm. **e** Confocal microscopy images of LXRα and ACTA2 in arterial walls of WT mice after ligation for 0, 7, 14, and 28 days. Scale bar, 20 µm. **f** Quantification of each lipid class in synthetic and contractile VSMCs. Lipid classes were expressed as μmol per g protein. **g** Heatmap of CEs between synthetic and contractile VSMCs. **h** ORO staining of WT VSMCs transducted with or without Ad-GFP-SM22α following with PDGF-BB and/or cholesterol treatment and quantification of positive ORO staining. Scale bar, 20 μm (*n* = 3). **i** M-mode and Doppler echocardiography images obtained from aortic arch and outflow tract of *Sm22α*^−/−^ mice transducted with AAV-GFP (*n* = 10) and AAV-SM22α (*n* = 10) fed Paigen diet for 12 weeks. *A*_s_: outflow tract and aortic diameter in systole; *A*_d_: outflow tract and aortic diameter in diastole. **j** Representative images of en face ORO-stained aortas and quantification of lesion areas (*n* = 6). **k** Representative immunofluorescence of LXRα (green) and quantification of cells with nuclear LXRα in the aortic sections from *Sm22α*^−/−^ mice transducted with AAV-GFP (*n* = 4) and AAV-SM22α (*n* = 4) fed Paigen diet for 24 weeks. Scale bar, 10 µm. Arrows indicated the distribution of LXRα. **l** Identification of SMC-derived foam cells within atherosclerotic lesion of *Sm22α*^−/−^ mice infected with AAV-GFP (*n* = 4) and AAV-SM22α (*n* = 4) fed Paigen diet for 24 weeks by CD68 (blue), ACTA2 (red), and Bodipy (green). Scale bar, 25 µm. Arrows indicated foam cells which were VSMCs-derived. Data and images are representative of at least three independent experiments. Data in **c** were analyzed by two-way ANOVA. Data in **f**, **h**, **j**, **k**, and **l** were analyzed by unpaired *t*-test. *p < 0.05; **p < 0.01; ***p < 0.001; ****p < 0.0001.

**Fig. 4:**
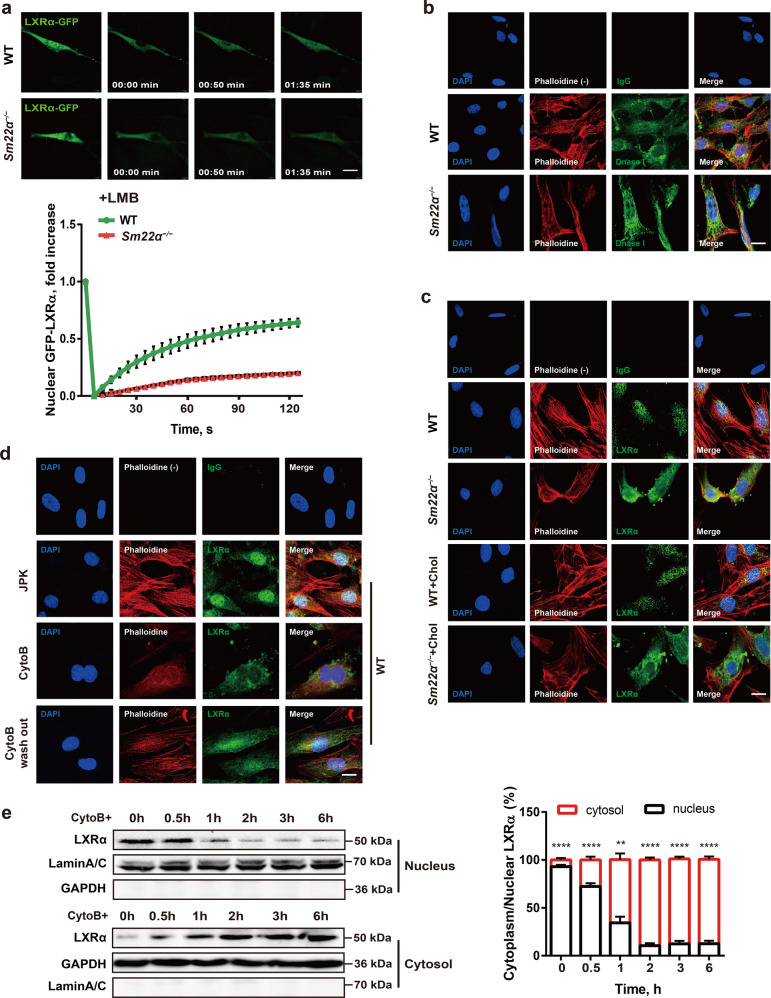
Nuclear import of LXRα is regulated by actin dynamics. **a** Fluorescence recovery after photobleaching (FRAP) studies with LXRα-GFP to measure nuclear import. Cells were pretreated with LMB. Decreased accumulation of nuclear fluorescence indicates a lower rate of nuclear import of LXRα-GFP in *Sm22α*^−/−^ VSMCs relative to WT controls (*n* = 25). **b** Representative images of F-actin (phalloidin, red) and G-actin (DnaseI, green) in WT and *Sm22α*^−/−^ VSMCs. Scale bar, 10 μm. **c**, **d** Representative images for F-actin (phalloidin, red) and LXRα (green) in WT and *Sm22α*^−/−^ VSMCs with cholesterol loading or not (**c**) and in WT VSMCs treated with JPK, CytoB and after CytoB washout (**d**). Scale bars, 10 µm. **e** Western blot analysis of cytoplasmic and nuclear LXRα in WT VSMCs treated with CytoB at different time points (*n* = 6). Data and images represent at least three independent experiments. Statistical analyses, unpaired *t*-test and Kruskal–Wallis rank sum test. **p < 0.01; ***p < 0.001; ****p < 0.0001.

**Fig. 5:**
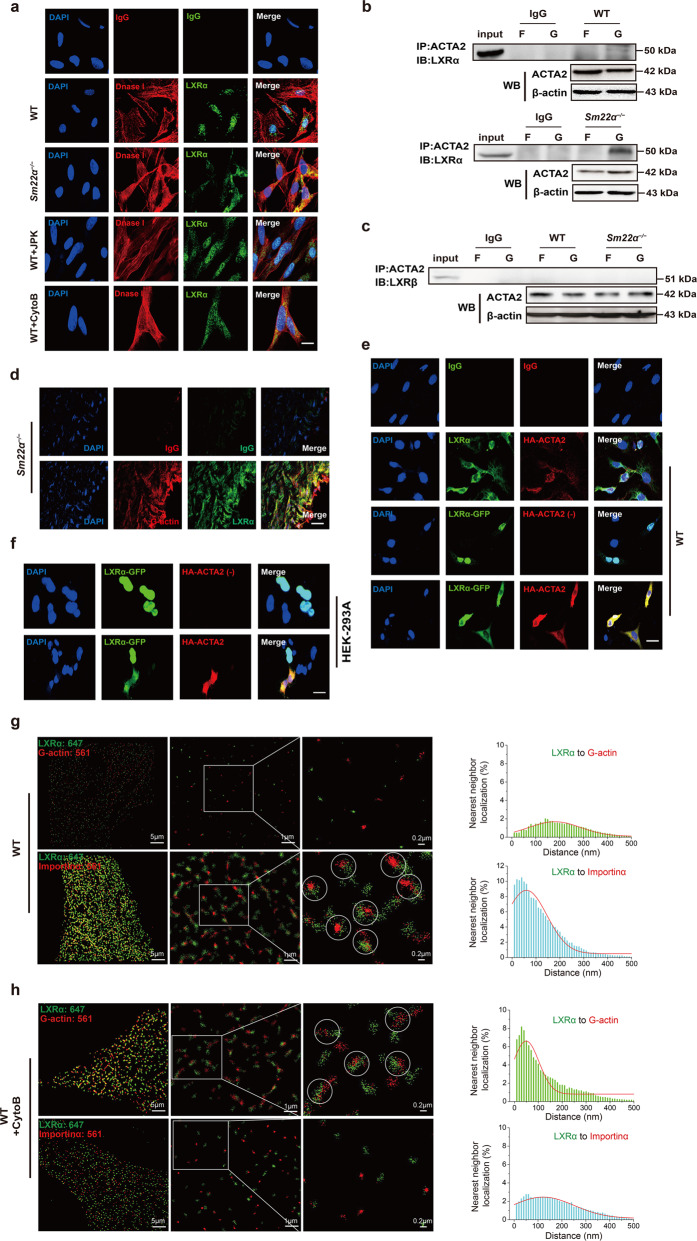

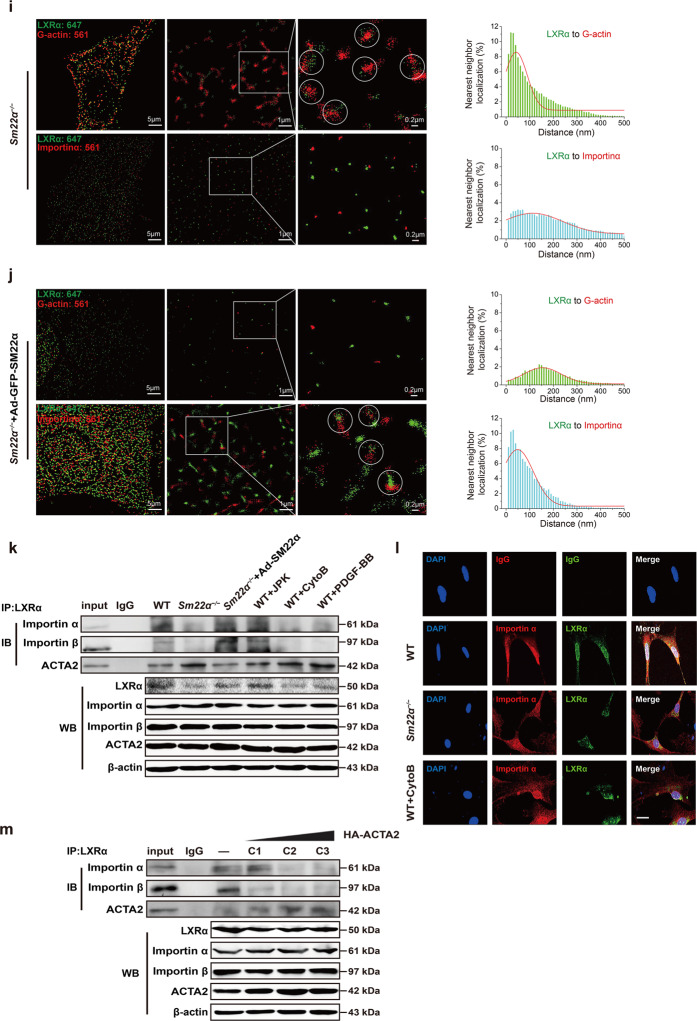
G-actin interacts with and retains LXRα in the cytoplasm, blocking LXRα binding to Importin α. **a** Double immunofluorescence staining for G-actin (DnaseI, red) and LXRα (green) in WT VSMCs accompanied with treatment of JPK or CytoB and also in *Sm22α*^−/−^ VSMCs. Scale bar, 10 μm. **b**, **c** Co-immunoprecipitation of ACTA2 and LXRα (**b**) and LXRβ (**c**), respectively, in F- and G-actin fractions of WT and *Sm22α*^−/−^ VSMCs (*n* = 3). **d** Double immunofluorescence staining of G-actin (Dnase1, red) and LXRα (green) or IgG in atherosclerotic lesion in aortic wall of *Sm22α*^−/−^ mice. Scale bar, 20 μm. **e** Representative immunofluorescence staining for endogenous LXRα (green) and LXRα-GFP (green) in WT VSMCs transfected with HA-ACTA2 (red, stained by anti-HA antibody) or not. Scale bar, 15 μm. **f** Representative immunofluorescence staining for LXRα-GFP (green) and HA-ACTA2 (red, stained by anti-HA antibody) in HEK-293A cells. Scale bar, 10 μm. **g**–**j** Two-color STORM images and quantification of the co-localization degree between LXRα and G-actin as well as Importin α in WT VSMCs with (**h**) or without (**g**) CytoB treatment and *Sm22α*^−/−^ VSMCs with (**j**) or without (**i**) Ad-GFP-SM22α infection (*n* > 10). **k** Co-immunoprecipitation of LXRα and Improtin α, Improtin β or ACTA2 in WT and *Sm22α*^−/−^ VSMCs with or without JPK, CytoB, PDGF-BB, and Ad-GFP-SM22α treatment (*n* = 3). **l** Double immunofluorescence staining for Importin α (red) and LXRα (green) in WT and *Sm22α*^−/−^ VSMCs as well as CytoB-treated WT VSMCs. Scale bar, 15 μm. **m** Co-immunoprecipitation of LXRα and Importin α, Improtin β or ACTA2 in WT VSMCs transfected with HA-ACTA2 of different concentration (*n* = 3). Data and images represent at least three independent experiments.

**Fig. 6:**
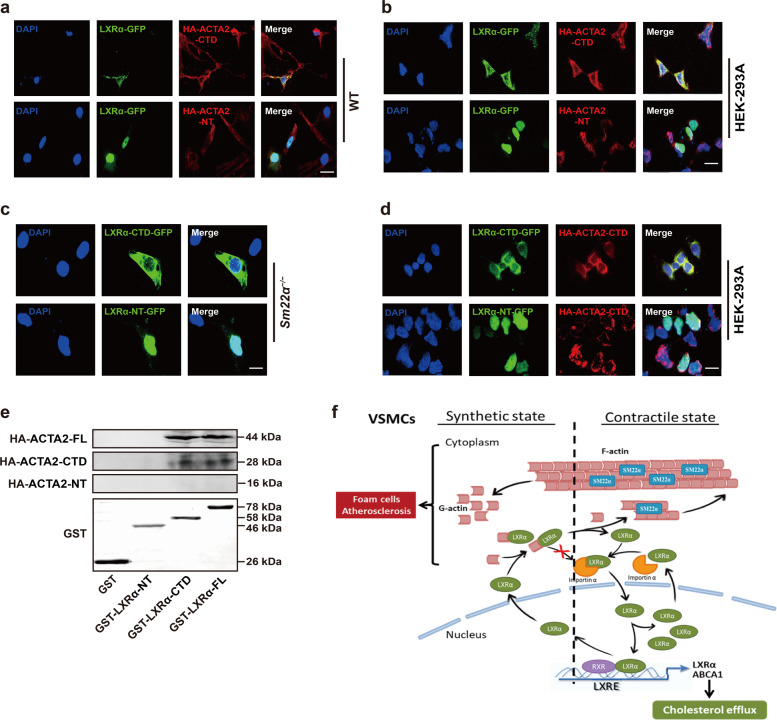
The C-terminal domain mediates interaction between G-actin and LXRα. **a** Representative immunofluorescence staining for LXRα-GFP (green) in WT VSMCs transfected with HA-ACTA2-CTD (red) or HA-ACTA2-NT (red). Scale bar, 15 μm. **b** Representative immunofluorescence staining for LXRα-GFP (green) and HA-ACTA2-CTD (red) or HA-ACTA2-NT (red) in HEK-293A cells. Scale bar, 15 μm. **c** LXRα-CTD-GFP (green) or LXRα-NT-GFP (green) was transfected into *Sm22α*^−/−^ VSMCs. Scale bar, 10 μm. **d** LXRα (-CTD, -NT)-GFP (green) and HA-ACTA2-CTD (red) were co-expressed in HEK-293A cells. Scale bar, 10 μm. **e** Interaction of HA-ACTA2 (-FL, -CTD, -NT) and GST-LXRα (-FL, -CTD, -NT) proteins analyzed by in vitro pull-down assay (*n* = 3). **f** Schematic representation of a working model which SM22α inhibits VSMC-derived foam cell formation by blocking actin-LXRα signaling ameliorating atherosclerosis. Data and images represent at least three independent experiments.

