# Supplementary material for: Smooth muscle 22 alpha protein inhibits VSMC foam cell formation by supporting normal LXRα signaling, ameliorating atherosclerosis
Source: Cell Death Dis. 2021 Oct 22;12(11):982. doi: 10.1038/s41419-021-04239-w (PMC8536684; doi:10.1038/s41419-021-04239-w)
Supplement: Supplementary file 1 — Supplementary Materials [file 41419_2021_4239_MOESM1_ESM.docx]

Supplementary Materials for

Smooth muscle 22 alpha protein inhibits VSMC-derived foam cell formation by supporting normal LXRα signaling, ameliorating atherosclerosis

Dan-Dan Zhang^1^†, Yu Song^1^†, Peng Kong^1^†, Xin Xu^1^, Ya-Kun Gao^1^, Yong-Qing Dou^3^, Lin Weng^1^, Xiao-Wei Wang^2^, Yan-Ling Lin^1^, Fan Zhang^1^, Hailin Zhang^4^, Mei Han^1^*

Corresponding author: Mei Han^1^*

Correspondence to: [hanmei@hebmu.edu.cn](mailto:hanmei@hebmu.edu.cn) (M.H.)

**This file includes:**

Materials and Methods

Supplementary Figs. 1-6

Supplementary Table

Captions for Movies S1-S2

**Materials and Methods**

**Reagents**

LXR agonist T0901317 (T090), rhodamine-phalloidine, recombinant human PDGF-BB were obtained from Abcam. Cyclodextrin (CD) cholesterol-complex, bodipy, oil red o (ORO), Cytochalasin B (Cyto B), jasplakinolide (JPK) and leptomycin B (LMB) were purchased from Sigma-Aldrich. Anti-DnaseI, anti-Importin α, anti-Importin β and anti-col1α were products from Santa Cruz Biotechnology. Antibodies were purchased from suppliers as follows: anti-SM22α (ab14106), anti-LXRα (ab176323), anti-LXRβ (ab28479), anti-ABCA1 (ab18180), anti-CD68 (ab955), anti-ACTA2 (ab124964) anti-GAPDH (ab181602), anti-phospho-(Ser/Thr) (ab17464) and anti-β-actin (ab6276) were from Abcam; anti-HA (51064-2-AP), anti-GST (HRP-66001), anti-RXR (21218-1-AP) were from Proteintech;.TRITC- or FITC-conjugated secondary antibodies were from KPL (Kirkegaard & Perry Laboratories, Inc). DyLight 649 AffiniPure goat anti-mouse antibody (E032610) was from EarthOx.

**Animals and treatments**

The *Sm22α*^−/−^ mice line (B6.129S6-Tagln^tm2(cre)Yec^/J) which has a Cre-recombinase gene inserted into the endogenous transgelin (SM22α) locus and *Ldlr*^−/−^ mice were purchased from the Jackson Laboratory[^1^](#_ENREF_7). *Sm22α*^−/−^ mice were generated by knocking-in the Cre-recombinase coding sequence into the endogenous SM22α gene locus via homologous recombination in embryonic stem (ES) cells. The targeting vector was generated in the pKO plasmid, which contains the PGKneo-poly(A) and pol2-DTA-poly(A) cassettes for positive and negative selection, respectively. The targeting vector contains a genomic fragment that includes 3 kb of the SM22α 5'-flanking sequence and the exon 1 sequence through the initiation codon. The Cre-recombinase coding sequence was inserted in-frame at the SM22α initiation site, which allows the Cre-recombinase driven by the endogenous SM22α promoter. The targeting vector also includes the 2.8 kb SM22α genomic fragment that spans exon1 through intron 1 sequence for homologous recombination. The targeting vector linearized by *Sal I* was transfected into 129S6SvEv/Tac embryonic stem cells through electroporation. G418-resistant colonies were analyzed for homologous recombination by Southern blot analysis after *EcoR I* digestion with a 5' genomic probe that contained sequences that reside outside the targeting vector. Two targeted clones were injected into C57BL/6 blastocysts to produce chimeras, which were crossed with C57BL/6 female mice to generate heterozygous SM22α-Cre knock-in (KI) mice. The chimera mice produced from both targeted ES clones show germline transmission. The heterozygous mice were inbred for getting homozygous mice and further characterization. Genotyping was performed using a combination of three-primers: GGCCCAGGGGTTGTCAAAATAGTC (common forward primer, 5’ to the SM22α initiation site); CTCCTCCAGCTCCTCGTCATACTTC (wild type-specific); and CGCCGCATAACCAGTGAAACAG (knock-in specific). Reaction products of 480 bp, 758 bp or both, represent the wild type, homozygous SM22α-CreKI or heterozygous SM22α-CreKI mice, respectively.

*Ldlr*^−/−^ mice were crossbred with *Sm22α*^−/−^ mice to obtain *Sm22α*^−/−^*Ldlr*^−/−^ mice. The resulting breeding offsprings were analysed for the presence of *Ldlr*^−/−^ and *Sm22α*^−/−^ genotype through tail tip DNA analysis.

All mice were housed in the temperature- and humidity-controlled facility, with standard laboratory chow (16% protein, 4% fat, 6% fiber) and ad libitum access to food and water.

At the age of 8-12 weeks, mice were randomly grouped and fed with Paigen diet (7.5% lard, 1.25% cholesterol, 0.5% sodium cholate, 0.125% choline chloride) for 8 or 12 and 24 weeks.

The Adeno-Associated Virus (AAV)-SM22α was constructed and packaged by Hanbio Biosciences (Shanghai, China). AAV-GFP was used as a control. *Sm22α*^−/−^ mice were randomly grouped and injected with AAV-GFP or AAV-SM22α respectively (1×10^12^ vg/mL) by tail vein. After 24 h, the mice were fed with Paigen diet for 24 weeks. Four weeks after AAV injection, the thoracic-abdominal aorta was taken for frozen section and the fluorescence was observed directly to determine the effect of transfection by GFP expression.

The investigators were blinded to the group allocation during the experiment and when assessing the outcome. All animal procedures conformed to the Guide for the Care and Use of Laboratory Animals published by the US National Institutes of Health (NIH Publication, 8th Edition, 2011) and was approved by the Institutional Animal Care and Use Committee of Hebei Medical University.

**Echocardiography**

Ascending aorta was imaged at 3 cm above the aortic valve from the parasternal long-axis view. Systolic aortic diameter (As) was measured at the point of maximal anterior motion of the ascending aorta (systole), and diastolic aortic diameter (Ad) was measured at the q wave on ECG (end diastole) using M-mode echocardiography. The mean of 5 measurements for diameter in sequential cardiac cycles was used for data analysis. Three elastic indices of aortic stiffness, namely aortic distensibility (D), stiffness index (β), and elastic modulus (Ep), were calculated as D=2(As-Ad)/[Ad(Ps-Pd)], β=ln(Ps/Pd)/[(As-Ad)/Ad], and Ep=(Ps-Pd)/[(As-Ad)/Ad], respectively, where Ps=systolic blood pressure, Pd=diastolic blood pressure, and ln=natural logarithm.

**Cell culture and treatment**

VSMCs were isolated from the thoracic aorta of wild-type (WT) and *Sm22α*^−/−^ mice and cultured in low glucose Dulbecco’s-modified Eagle’s medium (DMEM) (Invitrogen, US) supplemented with 20% fetal bovine serum (FBS, Gibco), 100 U/mL penicillin and 100 μg/mL streptomycin. The VSMCs were maintained at 37°C in a humidified atmosphere containing 5% CO_2_, and only passages 4 to 15 cells at 70-80% confluence were used in the experiments, except if stated otherwise. Human embryonic kidney (HEK) 293A cells were purchased from ATCC and maintained in high glucose DMEM containing 10% FBS.

Serum starvation was done by withdrawing serum and incubating the cells in 0.3% FBS for 24 h before stimulated with T0901317 (5 μmol/L), cyclodextrin (CD) cholesterol-complexes (10 μg/mL), Cytochalasin B (CytoB, 1 μmol/L), jasplakinolide (JPK, 50 μmol/L), leptomycin (LMB, 5 nmol/L), PDGF-BB (10 ng/mL) and infection with adenoviruses as well as transfection with plasmids.

**Adenovirus packaging and transduction**

Full-length cDNA of SM22α was cloned into pEGFP-C2, a mammalian expression vector and encoding a red-shifted variant of wild-type GFP, to generate pEGFP-SM22α. Then the fusions of SM22α plus GFP were subcloned into pAd/CMV/V5-DEST Gateway Vector (Invitrogen) to make the GFP tagged SM22α adenovirus Ad-GFP-SM22α, according to the manufacturer’s protocol. Ad-GFP was obtained the same to the above. All of these clones were verified by sequencing[^2^](#_ENREF_7). The VSMCs were infected with the above adenovirus (5×10^9^ pfu/mL) for 24 h, washed and incubated in serum-free medium without adenovirus for 24 h, then stimulated with or without cyclodextrin (CD)-cholesterol complex.

**Plasmids construction and transfection**

Mouse LXRα and its truncations (-NT and -CTD) cDNA[^3^](#_ENREF_7) were synthesized by Sangon Biotech (Shanghai, China) and cloned into pEGFP vector (Geneseed Biotech, Guangzhou, China). Meanwhile, mouse ACTA2-FL, ACTA2-NT and ACTA2-CTD cDNA were also synthesized by Sangon Biotech (Shanghai, China) and cloned into pCMV-HA vector (Geneseed Biotech, Guangzhou, China). All of these clones were verified by sequencing.

Transient transfection was carried out using Lipofectamine iMax Reagent (Invitrogen) according to manufacturer’s protocol. 2.5-5 μg of plasmid DNA was used per transfection reaction.

**SiRNA transfection**

The cultured VSMCs were grown to 50-60% confluence, and then transfected with specific duplex siRNA, siSM22α (5'-GCG UGA UUC UGA GCA AGU UUU-3' and 5'-AAC UUG CUC AGA AUC ACG CCA-3') or non-specific scrambled siRNA, siCon (5'-GCU AGA GUA GCG GUG AAU UCG UU-3' and 5'-CGA AUU CAC CGC UAC UCU AGC UU-3') using Lipofectamine iMax Reagent (Invitrogen) according to the manufacturer’s protocol. At 6-12 h after transfection, VSMCs were treated with cyclodextrin (CD)-cholesterol complex as mentioned.

**Oil red O (ORO) staining**

Aortas and cultured VSMCs were washed three times with PBS, fixed for 20 min in 4% paraformaldehyde and stained for 30 min in 0.3% Oil Red O. The cells or aortas were then washed three times with PBS and photographed with microscope. Lesion areas were quantified with Image Pro Plus (IPP) software.

**Lipid extraction**

VSMCs were treated by 10 ng/ml PDGF-BB (R&D) for 24 h to induce phenotype switching. Contractile VSMCs were prepared by treatment with 10 μM ATRA (J&K) at serum-starved state for 24 h. VSMC phenotypes were identified by western blot of phenotypic markers. Lipid were extracted from approximately 10^6^ cells using a modified version of the Bligh and Dyer’s method as described previously[^4^](#_ENREF_7).

**Lipid analyses**

Polar lipids were analyzed using an Exion UPLC system coupled with a triple quadrupole/ion trap mass spectrometer (6500 Plus Qtrap; SCIEX) as described previously[^4^](#_ENREF_7). Individual lipid species were quantified by referencing to spiked internal standards.

**Thin Layer Chromatography (TLC)**

TLC is a semi-qualitative analytical method that allows chemical compounds to be separated and partially identified in a complex mixture. 30 µL lipid solution extracted from aortas of WT and *Sm22α*^−/−^ mice fed Paigen diet were put in TLC plate (silica gel 60 F-254, Merk, Germany) and were separated using dichloromethane/methanol (97:3 v/v) and dichloromethane/ethyl acetate (90:10 v/v) as solvent systems for two-dimensional chromatography.

**Proteomics**

WT VSMCs were stimulated with 20 ng/mL PDGF-BB to induce a synthetic phenotype which was detected by specific marker. Cells were collected and proteins pellets were sonicated and digested as previously described. Acquired data was searched using MaxQuant^5^ version 1.5.8.3 against SwissProt human proteome downloaded on 25/10/2017, and later exported to R for analysis. Log2 transformed data was analyzed using limma package to identify significant proteins (p value (with and without adjustment) < 0.05, logFC > 1.5). Pathway enrichment analysis was carried out using the fgsea package using logFC as a ranking statistic and pathways from Reactome database[^6^](#_ENREF_8).

**Photobleaching experiments**

For FLIP and FRAP experiments, an Olympus microscope (Japanese) equipped with a 63× oil immersion objective was used. Cells were plated on a coverslip and mounted onto a glass slide with a depression containing culture media. Cells had been LMB-stimulated to inhibit nuclear export prior to the experiments. Relative increase in nuclear fluorescence after photobleaching of nuclear LXRα-GFP was computed by normalizing nuclear fluorescence intensity to pre-bleach values (*t*=0). Increased accumulation of nuclear fluorescence indicates a higher rate of nuclear import of LXRα-GFP. Cells were scanned two times before photobleaching by scanning the region of interest 80 times at 100% laser intensity of a 488 nm laser line. Single section images were then collected at 5 seconds intervals with laser power attenuated to 2% of the bleach intensity. The fluorescence intensity at the region of interest at each time point was normalized to the change in total fluorescence due to bleaching and imaging, as described above.

**Immunofluorescent staining**

Cells were fixed in 4% paraformaldehyde and permeabilized with 0.1%-0.5%Triton X-100 at room temperature for 20 minutes. Thereafter, cells were incubated with anti-SM22α, anti-LXRα, anti-LXRβ, anti-CD68, anti-DnaseI (labeled for G-actin), anti-ACTA2, anti-Importin α and anti-HA antibodies and further stained with phalloidin (labeled for F-actin, 1/1000 dilution) or bodipy (labeled for lipid) or appropriate DyLight 649-, TRITC- or FITC-conjugated secondary antibodies or Alexa Fluor 647 and Alexa Fluor 568 for STORM imaging. Confocal microscopy was performed with the Confocal Laser Scanning Microscope Systems (Leica).

**STORM imaging**

Cells were stained as described in immunofluorescent staining. Then the STORM imaging was performed with superresolution microscope Nikon N-STORM equipped with a 100× oil-immersion objective (numerical aperture 1.40) and an EM-CCD camera (Andor iXon, DV887-DCS-BV, Andor Technology ). Imaging was performed in PBS containing 1.5% β-Mercaptoethanol (Sigma), 5% glucose (Sigma), 0.8 mg/mL glucose oxidase (Sigma), and 40 µg/mL catalase (Sigma). A typical STORM image was generated from a sequence of about 30,000 image frames at a frame rate of 60 Hz. The duration of the acquisition was the same in all experiments. During imaging, continuous illumination of 647 nm laser (~0.8 kW/cm^2^) was used to excite fluorescence from Alexa-647 and switched them into the dark state. Continuous illumination of the 561 nm laser (~0.3 kW/cm^2^) was used to reactivate the fluorophores to the emitting state and the illumination power was controlled so that at any given instant, only a small, optically resolvable fraction of the fluorophores in the field of view were in the emitting state. SML fitting was conducted with the Nikon-integrated routine taking into account drift and chromatic aberrations. The recorded STORM movies were analyzed according to previously described methods[^7^](#_ENREF_9). Alexa 647 and 561 molecules were differentiated by the ratio of intensities detected in the short and long wavelength channels. Superresolution images were reconstructed from the molecular coordinates by depicting each location as a 2D Gaussian peak.

**Nearest-neighbor localization distance in STORM images**

The method of quantify the nearest-neighbor distance was performed as described previously[^8^](#_ENREF_3). Molecules lists of a pair of activator/reporter dye (Alexa 561/Alexa 647) from STORM images were exported from the N-STORM module of NIS-Elements in a separate .txt file, which contain corrected “X” and “Y” coordinates for each channel (color). The “X” and “Y” coordinates of these molecules was used as their precise locations in the images, and was input into a Matlab program to calculate the distances of each molecule with the other molecules from the same channel, or from the other channel(s). For each molecule, only the shortest distances in two channels (Alexa 561/Alexa 647) were saved, and these shortest distances were outputted in separate .txt files, which contain the shortest distances for the following pairs: Alexa 561 to Alexa 561, Alexa 561 to Alexa 647, Alexa 647 to Alexa 561, Alexa 647 to Alexa 647. Origin 9 were used to plot the distances from each .txt file into distribution histograms with bin size of 10 nm, which were then fit with Gaussian distribution.

**Live VSMC stiffness measurement using AFM**

Stiffness properties of VSMCs were performed using an Asylum AFM System (Model MFP-3D-BIO, Asylum Research, Santa Barbara, CA) mounted on an inverted microscope (Model IX81, Olympus America Inc.) as previous described[^9^](#_ENREF_4).

**RNA isolation and quantitative reverse transcription-PCR (qRT-PCR).**

Total RNA was extracted from cell cultures using TRIzol Reagent (Invitrogen) and treated with DNaseI to remove genomic DNA. A quantitative real-time reverse-transcriptase (RT)-PCR was performed with a Bio-Rad thermocycler and a SYBR green kit (Invitrogen) following manufacturer’s instructions. The relative mRNA expression was normalized to β-actin. Sequence-specific primers used were presented as following: SM22α (5'-AGG TGT GGC TGA AGA ATG GCG-3' and 5'-TCT TCG TGA CTC CAT AAT CCTC-3'), β-actin (5'-CAC CCG CGA GTA CAA CCT TC-3' and 5'-CCC ATA CCC ACC ATC ACA CC-3'), LDLR (5'-TCA GAC GAA CAA GGC TGT CC-3' and 5'-CCA TCT AGG CAA TCT CGG TCTC-3'), SR-BI (5'-AAA CAG GGA AGA TCG AGC CAG-3' and 5'-GGT CTG ACC AAG CTA TCA GGTT-3'), LXR (5'-AAC TGA AGC GGC AAG AAG AGG AAC-3' and 5'-TGT GGC AGG ACT TGA GGA GGTG-3'), ABCA1 (5'-TCA GTT TGA CGC CAT GAC AGAG-3' and 5'-ACC AAC CTT GCC AAC TTC CTT-3'), ABCG1 (5'-CTT TCC TAC TCT GTA CCC GAGG-3' and 5'-CGG GGC ATT CCA TTG ATA AGG-3'), LXRα (5'-CTC AAT GCC TGA TGT TTC TCCT-3' and 5'-TCC AAC CCT ATC CCT AAA GCAA-3'), LXRβ (5'-ATG TCT TCC CCC ACA AGT TCT-3' and 5'-GAC CAC GAT GTA GGC AGA GC-3'), Col1α (5'-GTG TGA TGG GAT TCC CTG GAC CTA-3' and 5'-CCG AGC TCC AGC TTC TCC ATC TT-3'), Eln (5'-TTG CTG ATC CTC TTG CTC AAC-3' and 5'-GCC CCT GGA TAA TAG ACT CCAC-3').

**Western blot and co-immunoprecipitation**

Lysates from cells were prepared with lysis buffer (1% Triton X-100, 150 mmol/L NaCl, 10 mmol/L Tris-HCl, pH 7.4, 1 mmol/L EDTA, 1 mmol/L EGTA, pH 8.0, 0.2 mmol/L Na_3_VO_4_, 0.2 mmol/L PMSF, and 0.5% NP-40). Equal amounts of protein (30-100 μg) were separated by 8% or 10% or 12% SDS-PAGE, and electro-transferred to a PVDF membrane. Membranes were blocked with 5% BSA (bovine serum albumin) for 2 h at room temperature, and incubated with specific antibodies as described above at 4°C overnight, and then with the HRP-conjugated secondary antibody (1:10000, Santa Cruz Biotechnology) for 1 h. The blots were evaluated with the Chemiluminescence plus Western blot analysis kit or ECL (enhanced chemiluminescence) detection system. These experiments were replicated three times.

For co-immunoprecipitation, WT and *Sm22α*^−/−^ VSMCs were lysed in a buffer composed of 50 mmol/L Tris-HCl, pH 7.6, 150 mmol/L NaCl, 1% NP-40, 10 mmol/L sodium phosphate, 10 mmol/L NaF, 1mM sodium orthovanadate, 2 mmol/L PMSF, 10 μg/mL aprotinin, 10 μg/mL leupeptin, and 10 μg/mL pepstatin. After centrifugation, the supernatants were first precleared with 20 μL protein A/G-agarose (Santa Cruz) to reduce nonspecific binding and immunoprecipitated with anti-RXRα or anti-pSer antibodies at 4°C overnight, followed by incubation with protein A/G-agarose beads for 2 h. After 24 h incubation, the immune complexes were centrifuged, and washed four times with ice-cold lysis buffer. The immunoprecipitated protein was further analyzed by Western blot as described above.

**F- and G-actin fractionation**

Medium was removed and cells were collected in 1.5 mL of actin lysis buffer (20 mmol/L HEPES, pH 7.9, 50 mmol/L NaCl, 1 mmol/L EDTA, 0.5% Triton X-100, and complete protease inhibitors from Roche); 0.5 mL samples of medium were removed for input samples. Remaining lysates were centrifuged for 2 h at 4 degrees at 100,000× *g*. Supernatants were transferred to a new tube (the G-actin fraction). The pellet (the F-actin fraction) was resuspended in 1 mL of actin lysis buffer and sonified (Branson sonifier; 5 × 10 s, duty cycle constant, output control 3). Samples were resolved on 10% SDS-PAGE and tested by anti-ACTA2 antibody.

**Glutathione S-transferase (GST) pull-down assay**

For the interaction of LXRα and ACTA2 or their truncations in vitro, GST, GST-LXRα-FL, GST-LXRα-NT and GST-LXRα-CTD fusion proteins were produced by BL21 E.coli under induction by isopropyl thio-β-D-galactoside (IPTG) at 25°C. Proteins were purified by affinity absorption using glutathione-Sepharose 4B beads (Amersham Pharmacia Biotech). The recombinant GST, GST-LXRα-FL, GST-LXRα-NT and GST-LXRα-CTD proteins on the glutathione beads were incubated with total cell lysates of HEK293 cells transfected with HA-ACTA2-FL, HA-ACTA2-CTD and HA-ACTA2-CTD at 4°C overnight followed by extensive washing. Proteins on the beads were resolved on 10% SDS-PAGE and visualized by immunoblotting with anti-SM22α and anti-TRAF6 antibodies.

**Chromatin immunoprecipitation (ChIP) assay**

ChIP assays were performed using the PierceTM Agarose ChIP Assay Kit (Thermo Scientific) according to manufacture instruction. VSMCs were fixed in 1% formaldehyde for 10 min to cross link proteins with DNA. The cross-linked chromatin was then prepared and sonicated to an average size of 200-600 bp. The samples were precleared with Dynabeads Protein G (Life technology) for 30 min at 4°C. The DNA fragments were immunoprecipitated overnight at 4°C with anti-LXRα and normal rabbit IgG (Santa Cruz) antibodies. The precipitated DNA was recovered via phenol/chloroform extraction, and LXRα binding site of *abca1* (Forward primer: 5'-CGA GCT TTT CCC CTT TCC TC-3', Reverse primer: 5- CCT TTT ATA GAT TCG GCT GTGC-3') and *col1α* (Forward primer: 5'-CCA CCC CAC CCG CAC TG-3', Reverse primer: 5'-TCC CGC CTC CTC CAA ACC-3') gene promoter region was amplified by qPCR. Each experiment was replicated at least three times.

**Data analysis**

Statistical analysis was performed using GraphPad Prism (GraphPad Software Inc., La Jolla, CA, USA). Data from at least three independent tests performed in triplicates are presented as the means ± SEM. Error bars in the scatterplots and the bar graphs represent SEM. For data with normal distribution, ANOVA was employed for statistical analysis when more than two groups were compared, and a two-tailed Student’s t test was used when comparison between two groups was performed. Nonparametric Kruskal-Wallis rank sum test was used for continuous variable with non-normally distributed data. One representative experimental result was selected to display in present study from three or more independent experiments. Statistical significance was defined as *p* < 0.05.

**References**

1. Zhang J, Zhong W, Cui T, Yang M, Hu X, *et al*. Generation of an adult smooth muscle cell-targeted Cre recombinase mouse model. *Arterioscler* Thromb Vasc Biol **26**, 23-24 (2006).

2. Shu YN, Zhang F, Bi W, Dong LH, Zhang DD, Chen R, et al. SM22alpha inhibits vascular inflammation via stabilization of IkappaBalpha in vascular smooth muscle cells. J Mol Cell Cardiol **84**, 191-199 (2015).

3. Li X, Zhang S, Blander G, Tse JG, Krieger M, Guarente L. SIRT1 deacetylates and positively regulates the nuclear receptor LXR. Mol Cell **28**, 91-106 (2007).

4. Song J-W, Lam SM, Fan X, Cao W-J, Wang S-Y, Tian H, et al. Omics-Driven Systems Interrogation of Metabolic Dysregulation in COVID-19 Pathogenesis. Cell Metab **32**, 188-202 (2020).

5. Cox J, Mann M. MaxQuant enables high peptide identification rates, individualized p.p.b.-range mass accuracies and proteome-wide protein quantification. Nat Biotechnol **26**, 1367-1372 (2008).

6. Jassal B, Matthews L, Viteri G, Gong C, Lorente P, Fabregat A, et al. The reactome pathway knowledgebase. Nucleic Acids Res **48**, 498-503 (2020).

7. Dudok B, Barna L, Ledri M, Szabo SI, Szabadits E, Pinter B, et al. Cell-specific STORM super-resolution imaging reveals nanoscale organization of cannabinoid signaling. Nat Neurosci **18**, 75-86 (2015).

8. Zhang J, Carver CM, Choveau FS, Shapiro MS. Clustering and Functional Coupling of Diverse Ion Channels and Signaling Proteins Revealed by Super-resolution STORM Microscopy in Neurons. Neuron **92**, 461-478 (2016).

9. Sanyour HJ, Li N, Rickel AP, Torres HM, Anderson RH, Miles MR, et al. Statin-mediated cholesterol depletion exerts coordinated effects on the alterations in rat vascular smooth muscle cell biomechanics and migration. J Physiol **598**, 1505-1522 (2020).

**Supplementary Figure Legends**

**Supplementary Fig. 1 Impaired SM22α expression is associated with development of atherosclerosis.** **a-c** Serum TC (**a**), LDL-C (**b**) and TG (**c**) levels of WT and *Sm22α*^−/−^ mice fed Paigen diet were monitored. (n=12) **d** TLC analysis for cholesterol content in aortic wall of WT and *Sm22α*^−/−^ mice fed paigen diet. FC: free cholesterol; CE: cholesterol ester. (n=3) **e** ORO and HE staining of aortic sinus and aortic cross sections in WT and *Sm22α*^−/−^ mice with or without *Ldlr*^−/−^ background fed Paigen diet for 8 and 12 weeks respectively. Arrows indicated the injured elastic lamina. **f** Stiffness parameters (elastic modulus, stiffness index, reverse/forward flow ratio and distensibility) obtained from aortic arch and outflow tract of WT and *Sm22α*^−/−^ mice transducted with or without AAV-GFP and AAV-SM22α fed Paigen diet for 12 and 24 weeks. (n=15) **g-h** Masson trichrome (**g**), picosirius red (**h**) staining and quantification on aortic arch and outflow tract in WT and *Sm22α*^−/−^ mice after Paigen diet. (n=6) **i** Immunohistochemical staining of Col1α and Eln in aortas of WT and *Sm22α*^−/−^ mice after Paigen diet. (n=6) **j-k** The expression of LXRα (**j**) and ABCA1 (**k**) at mRNA and protein levels in the aortas of WT and *Sm22α*^−/−^ mice fed chow diet or Paigen diet. (n=3) Data and images are representative of at least three independent experiments. Data in **a-c** were analyzed by Kruskal-Wallis rank sum test. Data in **f**, **g**, **h** and **i** were analyzed by unpaired *t* test. Data in **j** and **k** were analyzed by one-way ANOVA. *, *p*<0.05; **, *p* <0.01; ***, *p* <0.001; ****, *p* <0.0001.

**Supplementary Fig. 2 Expression and activity of LXRα is abnormal in *Sm22α*^−/−^ VSMCs. a** qRT-PCR and Western blot analysis of LXRα and LXRβ expression in WT and *Sm22α*^−/−^ VSMCs treated with cholesterol for 0, 12, 24, 48 and 72 h respectively. (n=3) **b** Western blot and quantification analysis for expression of LXRα in the cytosolic and nuclear fractions from WT and *Sm22α*^−/−^ VSMCs. (n=6) **c-e** WT VSMCs were transfected with siCon and siSM22α. (**c**) qRT-PCR and Western blot analysis of ABCA1 expression in WT VSMCs with or without cholesterol loading. (n=3) (**d**) ORO staining and quantification of positive ORO-stained cells following with cholesterol loading at different time points. Scale bar, 20 μm. Arrows indicated the positive ORO-stained VSMCs. (n=3) (**e**) Bodipy staining for lipid accumulation in VSMCs. Scale bar, 10 μm. (n=3) **f** ChIP and RT-PCR detected LXRα binding to *col1α* promoter in *Sm22α*^−/−^ VSMCs infected with Ad-GFP and Ad-GFP-SM22α. (n=3) **g** The mRNA and protein levels of Col1α in *Sm22α*^−/−^ VSMCs infected with Ad-GFP and Ad-GFP-SM22α. (n=3) Immunoblots and confocal microscopy images are representative of at least three independent experiments. Data in **a** were analyzed by Kruskal-Wallis rank sum test. Data in **b**-**e** were analyzed by one-way ANOVA. Data in **f** and **g** were analyzed by unpaired *t* test. *, *p*<0.05; **, *p* <0.01; ***, *p* <0.001; ****, *p* <0.0001.

**Supplementary Fig. 3 Targeting SM22α supports normal LXRα signaling and ameliorates atherosclerosis. a** GO analysis of proteomic between synthetic and contractile VSMCs annotates the biological process and clusters the modules of genes. The top 20 cluster ranked by *p* value was shown. **b** Western blot of markers for contractile and synthetic phenotypes of VSMCs used in lipidomics. **c** Representative immunofluorescence of GFP and SM22α in the aortic sections from *Sm22α*^−/−^ mice transducted with or without AAV-GFP and AAV-SM22α. (n=6) **d** Stiffness parameters (elastic modulus, stiffness index, reverse/forward flow ratio and distensibility) obtained from aortic arch and outflow tract of WT and *Sm22α*^−/−^ mice transducted with or without AAV-GFP and AAV-SM22α fed Paigen diet for 12 and 24 weeks. (n=6) **e** Immunohistochemical and HE, ORO staining of aortic cross sections in *Sm22α*^−/−^ mice infected with AAV-GFP and AAV-SM22α. **f** qRT-PCR analysis of cholesterol efflux genes (LXRα, ABCA1) and sclerosis related genes (Col1α, Eln) as well as LXRα-related inflammatory genes (MCP-1, MMP2, MMP9, VCAM-1, ICAM-1) in aortas of *Sm22α*^−/−^ mice transducted with AAV-GFP and AAV-SM22α. Data and images are representative of at least three independent experiments. Data were analyzed by unpaired *t* test. *, *p*<0.05; **, *p* <0.01; ***, *p* <0.001; ****, *p* <0.0001.

**Supplementary Fig. 4 Nuclear import of LXRα is regulated by actin dynamics. a** Immunoprecipitation analysis for phosphorylation of LXRα in WT and *Sm22α*^−/−^ VSMCs. (n=3) **b** Co-immunoprecipitation analysis for the interaction of LXRα and RXRα in WT and *Sm22α*^−/−^ VSMCs. (n=3) **c** GO analysis annotates the biological process and clusters the modules of genes. The top 20 cluster ranked by *p* value was shown. **d** Ratio of F-/G-actin was measured and quantified by Western blotting after fractionation of F- and G-actin in WT and *Sm22α*^−/−^ VSMCs under different conditions. (n=3) **e** Docking analysis revealed the 3D structural basis for possible interaction between G-actin and LXRα. Immunoblots images are representative of at least three independent experiments. Data are expressed as mean ± SEM of three independent experiments. Data were analyzed by two-way ANOVA. ***, *p* <0.001; ****, *p* <0.0001.

**Supplementary Fig. 5 G-actin directly interacts with and retains it in the cytoplasm.** **a** Confocal microscopy images of HA-ACTA2 (green, labeled by anti-HA antibody) and G-actin (red, labeled by DnaseI) in WT VSMCs transfected with HA-ACTA2. Scale bar, 10 µm. **b** Western blot analysis for expression of LXRα in the cytosolic and nuclear fractions from WT and *Sm22α*^−/−^ VSMCs in different conditions. (n=3) Immunoblots and confocal microscopy images are representative of at least three independent experiments.

**Supplementary Fig. 6 The C-terminal domain mediates interaction between G-actin and LXRα. a** Two structural domains of ACTA2 with HA-tagged truncated N-terminus (HA-ACTA2-NT, aa.1-140) and C-terminal domains (HA-ACTA2-CTD, aa.141-377) were reconstructed. **b** Two truncated mutants of LXRα-N-terminus (NT, aa.1-170) that contained the DNA binding domain (DBD) and 3 nuclear localization sequences (NLSs) (NLS1, 2 and 4) and LXRα-C-terminal domains (CTD, aa.171-445) that included the hinge region, one NLS (NLS3) and the putative ligand binding domain were constructed.

**Supplementary Table. Proteins identified in specific bands by MS may interact with LXRα.**

| Protein | IgG Peptide | LXRα Peptide | Protein | IgG Peptide | LXRα Peptide |
| --- | --- | --- | --- | --- | --- |
| ZKSCAN2 | 0 | 1 | MGST1 | 0 | 1 |
| ANXA2 | 0 | 7 | HSPA8 | 0 | 4 |
| LGALS1 | 0 | 4 | AHNAK | 0 | 3 |
| IGHG | 0 | 7 | P4HB | 0 | 4 |
| ACTA2 | 0 | 9 | HSPE1 | 0 | 1 |
| VIM | 0 | 13 | ATP5F1B | 0 | 4 |
| ACTB | 0 | 8 | HIST2H2BB | 0 | 1 |
| LC | 0 | 4 | HNRNPF | 0 | 1 |
| ANXA1 | 0 | 6 | KRT77 | 0 | 2 |
| ACTA1 | 0 | 5 | HSP90B1 | 0 | 5 |
| S100A11 | 0 | 1 | YBX1 | 0 | 1 |
| NOP56 | 0 | 3 | IGHG1 | 0 | 2 |
| SEC61B | 0 | 1 | TM4SF1 | 0 | 1 |
| HSPA5 | 0 | 7 | MDH2 | 0 | 1 |
| HIST1H1T | 0 | 1 | ATP5C1 | 0 | 1 |
| HSPA9 | 0 | 3 | SPECC1 | 0 | 1 |
| PDIA3 | 0 | 2 | CALR | 0 | 2 |
| KRT1 | 3 | 2 | SNX19 | 0 | 1 |
| ALB | 0 | 2 | SERPINH1 | 0 | 2 |
| ATP5A1 | 0 | 1 | HSPD1 | 0 | 2 |
| HNRNPA3 | 0 | 1 | TNRC18 | 0 | 1 |
| LMNA | 0 | 2 | SLC7A5 | 0 | 1 |
| KRT17 | 0 | 1 | PCDHB13 | 0 | 1 |
| CANX | 0 | 1 | PLEC | 0 | 2 |
| IGKV4-61 | 2 | 1 | IGKC | 2 | 3 |
| MYL6 | 2 | 1 | GM10481 | 1 | 3 |
| IGKV6-13 | 1 | 1 | IGH | 4 | 4 |
| PRSS1 | 1 | 1 | IGHV1-76 | 1 | 2 |
| KRT10 | 3 | 3 | KRT14 | 2 | 2 |
| KRT2 | 3 | 3 | KRT16 | 2 | 2 |
| MYH9 | 8 | 1 | EEF1A1 | 1 | 2 |
| KRT5 | 2 | 3 | STX1A | 1 | 3 |
| TMOD3 | 1 | 1 | KRT42 | 1 | 1 |
| KRT78 | 1 | 1 | ITPRID2 | 1 | 3 |
| KRT76 | 1 | 1 | RBBP8 | 1 | 2 |
| OVGP1 | 1 | 1 | RIK | 1 | 1 |

**Caption for Movie S1**: Fluorescence recovery after photobleaching (FRAP) studies with LXRα-GFP to measure nuclear import in WT VSMCs.

**Caption for Movie S2**: FRAP studies with LXRα-GFP to measure nuclear import in SM22α KO VSMCs.
