# Supplementary figures and images for: Smooth muscle 22 alpha protein inhibits VSMC foam cell formation by supporting normal LXRα signaling, ameliorating atherosclerosis
Source: Cell Death Dis. 2021 Oct 22;12(11):982. doi: 10.1038/s41419-021-04239-w (PMC8536684; doi:10.1038/s41419-021-04239-w)

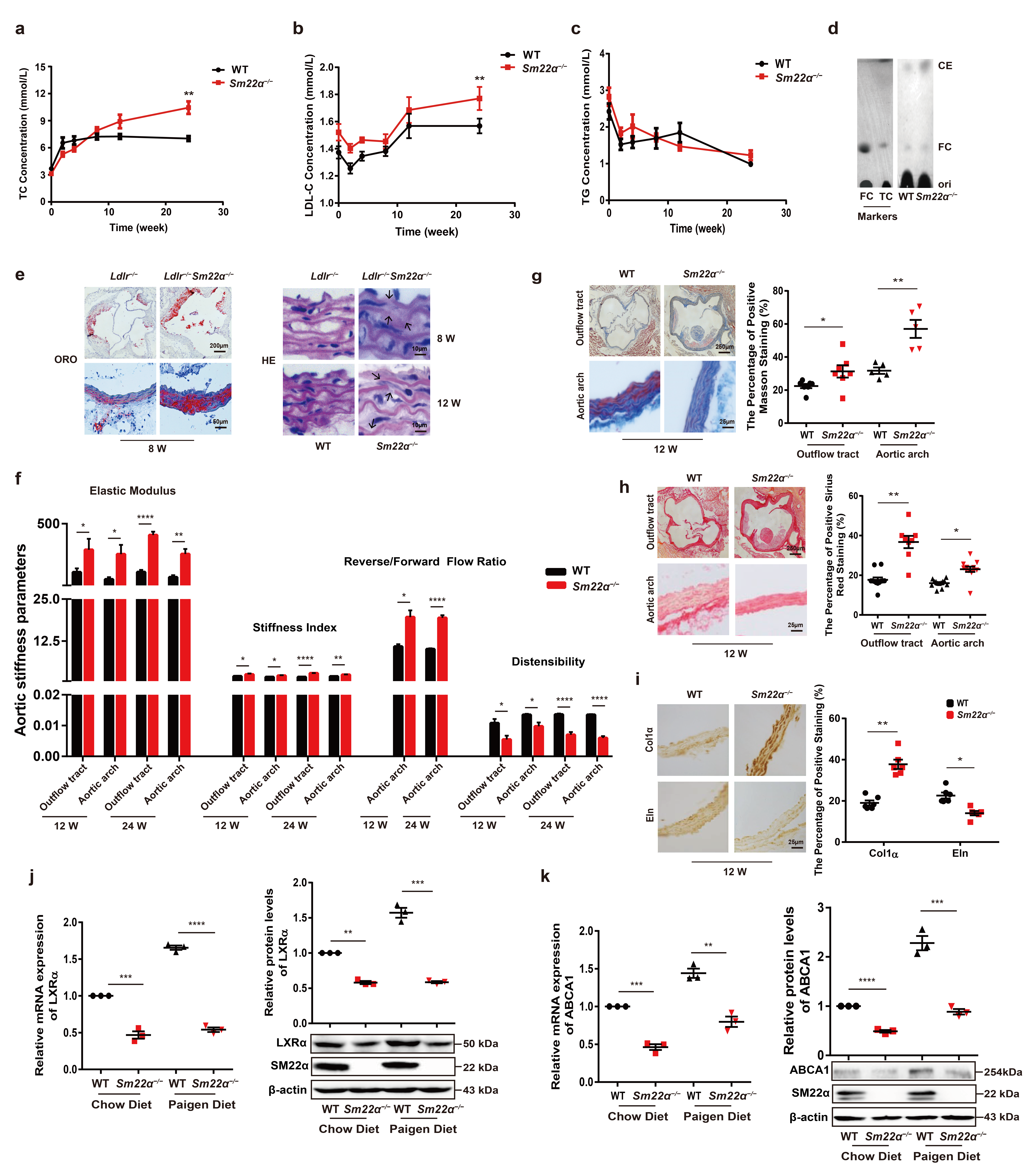

Supplement: Supplementary file 2 — Supplementary figure 1 [file 41419_2021_4239_MOESM2_ESM.png]

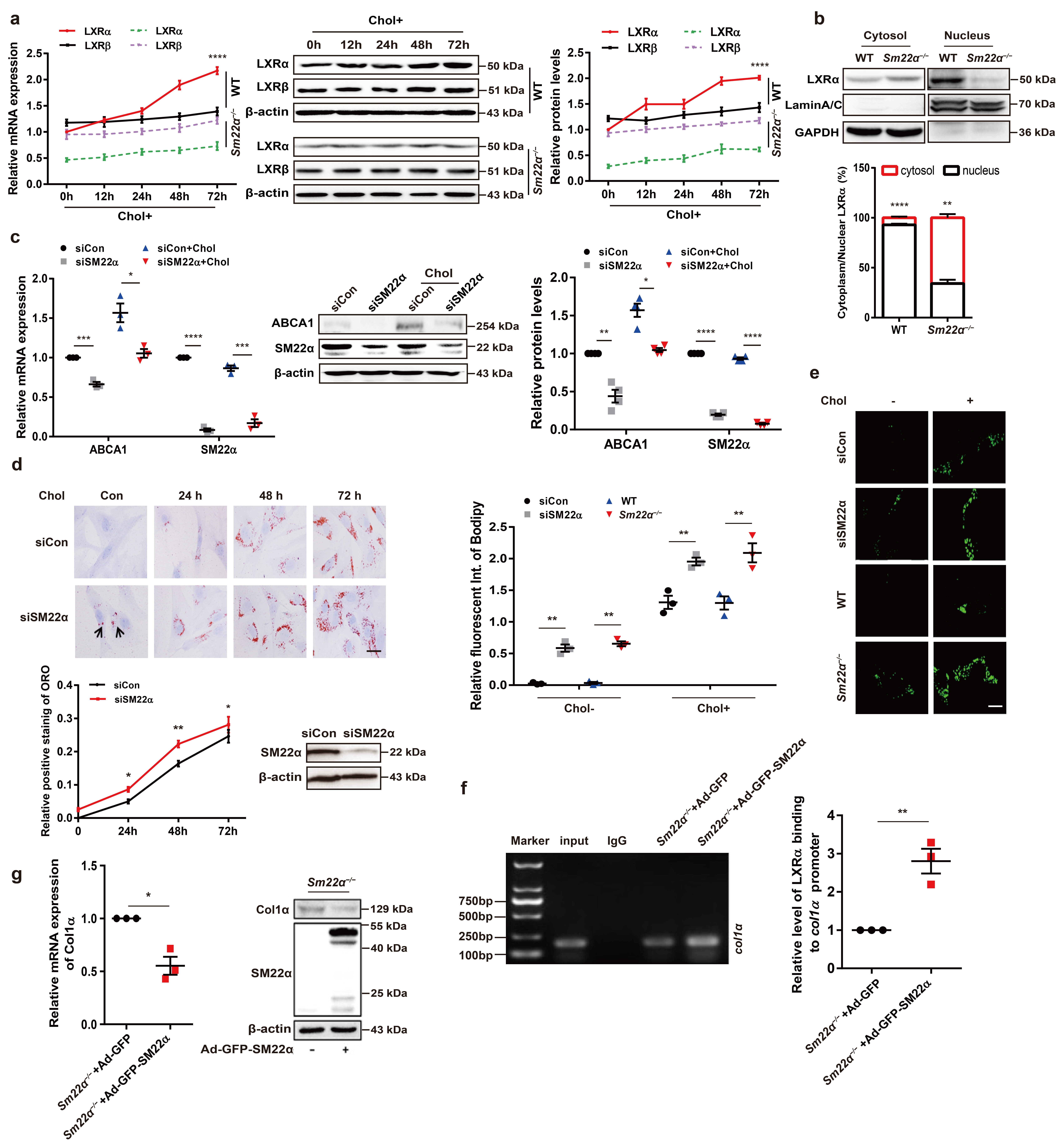

Supplement: Supplementary file 3 — Supplementary figure 2 [file 41419_2021_4239_MOESM3_ESM.png]

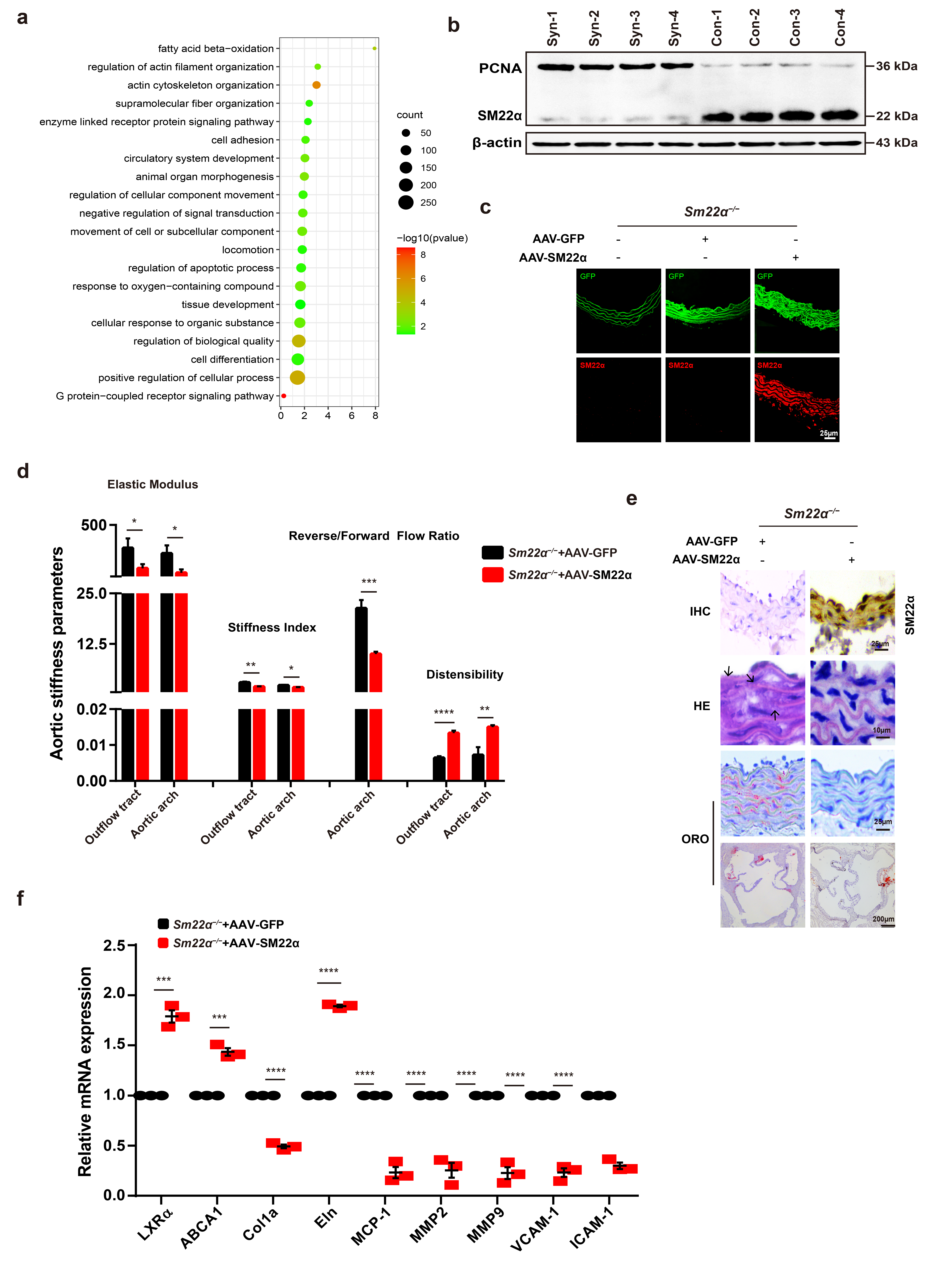

Supplement: Supplementary file 4 — Supplementary figure 3 [file 41419_2021_4239_MOESM4_ESM.png]

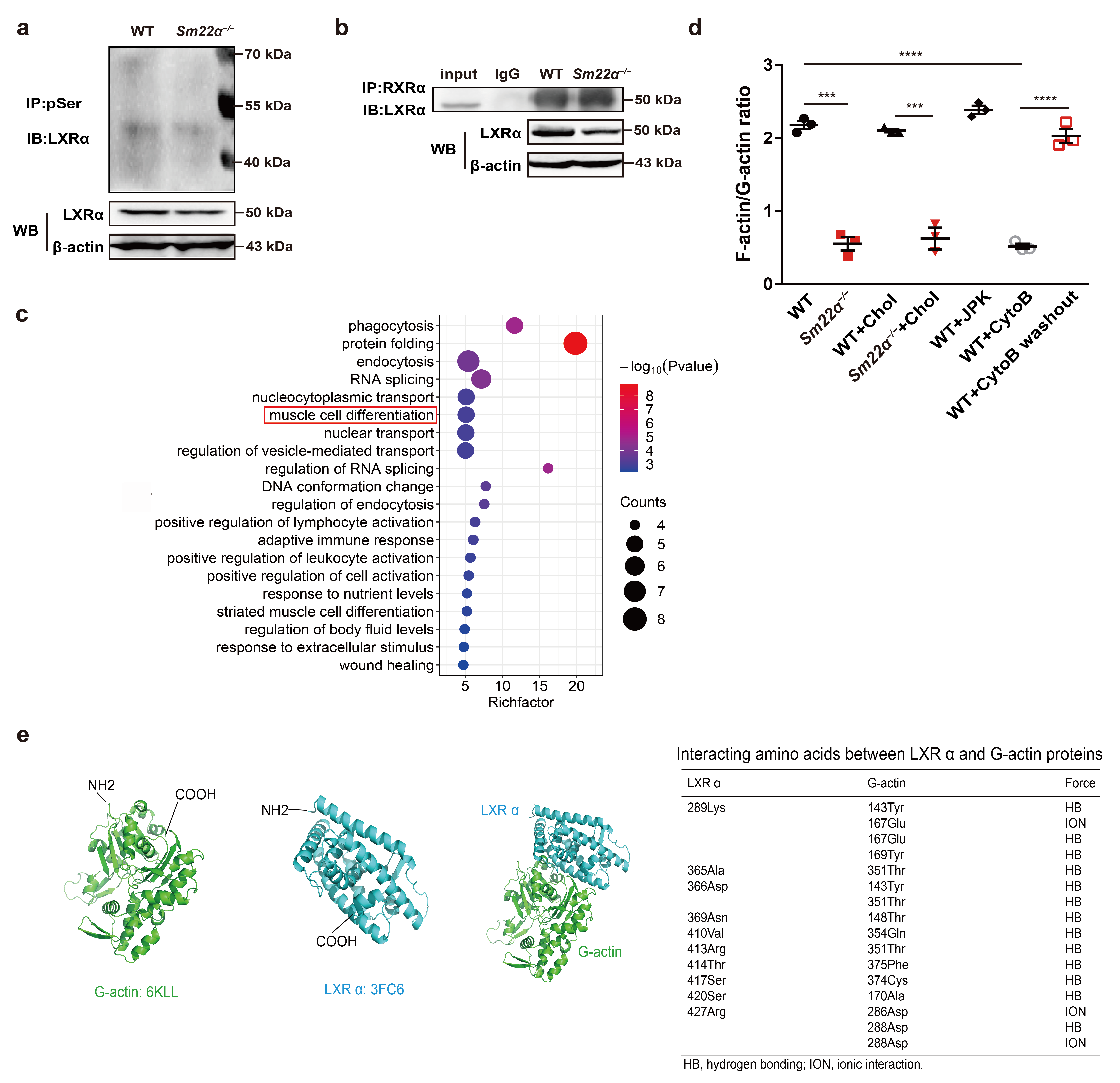

Supplement: Supplementary file 5 — Supplementary figure 4 [file 41419_2021_4239_MOESM5_ESM.png]

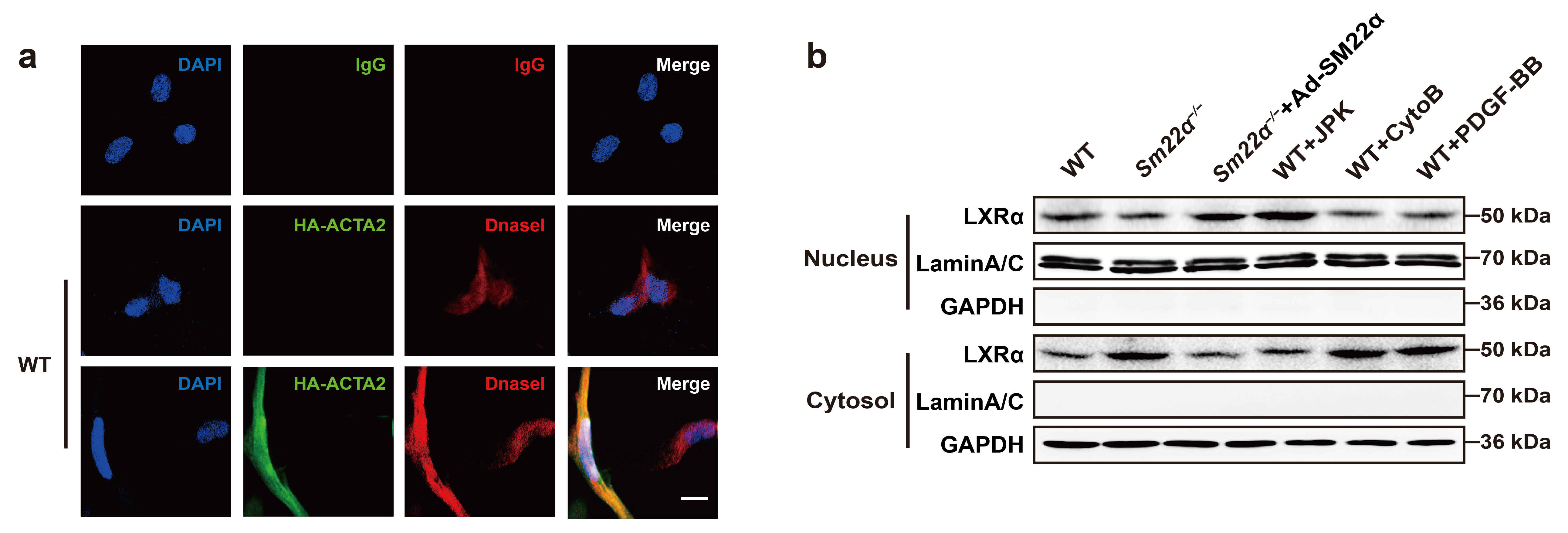

Supplement: Supplementary file 6 — Supplementary figure 5 [file 41419_2021_4239_MOESM6_ESM.png]

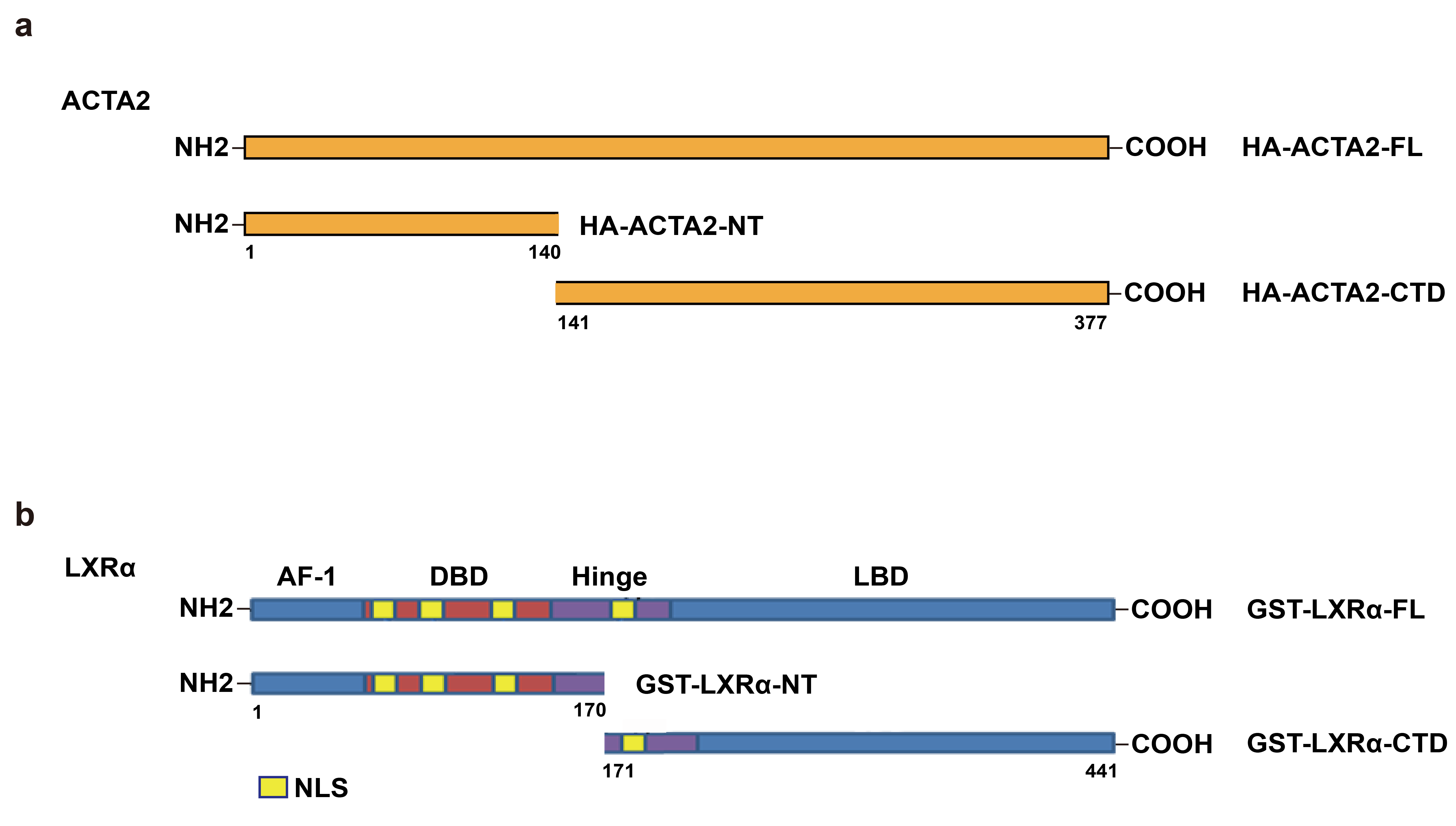

Supplement: Supplementary file 7 — Supplementary figure 6 [file 41419_2021_4239_MOESM7_ESM.png]
